# A high-coverage shRNA screen identifies TMEM129 as an E3 ligase involved in ER-associated protein degradation

**DOI:** 10.1038/ncomms4832

**Published:** 2014-05-08

**Authors:** Michael L. van de Weijer, Michael C. Bassik, Rutger D. Luteijn, Cornelia M. Voorburg, Mirjam A.M. Lohuis, Elisabeth Kremmer, Rob C. Hoeben, Emily M. LeProust, Siyuan Chen, Hanneke Hoelen, Maaike E. Ressing, Weronika Patena, Jonathan S. Weissman, Michael T. McManus, Emmanuel J.H.J. Wiertz, Robert Jan Lebbink

**Affiliations:** 1 Medical Microbiology, University Medical Center Utrecht, 3584CX Utrecht, The Netherlands; 2 Department of Cellular and Molecular Pharmacology, California Institute for Quantitative Biomedical Research, Howard Hughes Medical Institute, University of California, San Francisco, California 94158, USA; 3 Helmholtz Zentrum München, German Research Center for Environmental Health, Institute of Molecular Immunology, 81377 Munich, Germany; 4 Department of Molecular Cell Biology, Leiden University Medical Center, 2333ZC Leiden, The Netherlands; 5 Genomics Solution Unit, Agilent Technologies Inc., Santa Clara, California 95051, USA; 6 Department of Microbiology and Immunology, University of California, San Francisco, California 94143, USA; 7 These authors contributed equally to this work; 8 Present address: Department of Genetics, Stanford University, California 94305, USA; 9 Present address: Twist Bioscience, San Francisco, California 94158, USA; 10 Present address: Carnegie Institution for Science, Department of Plant Biology, Stanford, California 94305, USA

## Abstract

Misfolded ER proteins are retrotranslocated into the cytosol for degradation via the ubiquitin–proteasome system. The human cytomegalovirus protein US11 exploits this ER-associated protein degradation (ERAD) pathway to downregulate HLA class I molecules in virus-infected cells, thereby evading elimination by cytotoxic T-lymphocytes. US11-mediated degradation of HLA class I has been instrumental in the identification of key components of mammalian ERAD, including Derlin-1, p97, VIMP and SEL1L. Despite this, the process governing retrotranslocation of the substrate is still poorly understood. Here using a high-coverage genome-wide shRNA library, we identify the uncharacterized protein TMEM129 and the ubiquitin-conjugating E2 enzyme UBE2J2 to be essential for US11-mediated HLA class I downregulation. TMEM129 is an unconventional C4C4-type RING finger E3 ubiquitin ligase that resides within a complex containing various other ERAD components, including Derlin-1, Derlin-2, VIMP and p97, indicating that TMEM129 is an integral part of the ER-resident dislocation complex mediating US11-induced HLA class I degradation.

## Abstract (web summary)

The human cytomegalovirus protein US11 downregulates host immune responses by redirecting HLA class I molecules for endoplasmic reticulum-associated protein degradation. Using a high-coverage genome-wide shRNA screen, the authors identify TMEM129 as an E3 ubiquitin ligase essential for this process.

## Graphical abstract

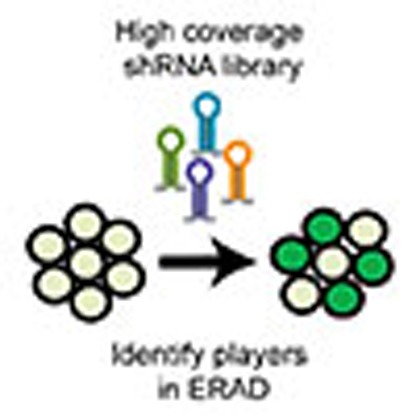

In the ER, newly synthesized proteins undergo a quality check by chaperones, which attempt to induce proper folding^1^. Failure to do so may result in protein accumulation and aggregation in the ER, thereby compromising protein and cellular homeostasis^2^,^3^. To prevent this, terminally misfolded luminal and transmembrane ER proteins are targeted for ubiquitin-dependent degradation by the proteasome in the cytosol^4^, a process called ER-associated protein degradation (ERAD)^5^,^6^. This process depends on retrograde transport, or dislocation, of proteins into the cytosol via a reaction that is facilitated by a multiprotein complex that combines several functions essential for ERAD, such as recognition, guidance, ubiquitination, dislocation and deglycosylation of the substrate^7^,^8^. ERAD is not only used for removal of misfolded proteins but also for physiologically regulated proteolysis of ER-resident proteins^9^,^10^.

Human cytomegalovirus (HCMV) encodes several proteins that impair the HLA class I antigen presentation pathway^11^, thus avoiding detection of infected cells by cytotoxic T lymphocytes^12^. In particular, HCMV US11 exploits ERAD to induce rapid dislocation of newly synthesized HLA class I heavy chains (HCs) from the ER into the cytosol, where the HCs are subsequently degraded via the ubiquitin–proteasome system^13^. The ER luminal domain of US11 is required for interaction with HLA class I, while the transmembrane domain of US11 is essential for interaction with Derlin-1 (refs 14, 15, 16). In this way, US11 recruits the HLA class I molecule to the dislocation complex, which besides Derlin-1 (refs 14, 17) contains VIMP^17^, the AAA ATPase p97 (refs 17, 18), Derlin-2 (ref. 19) and SEL1L^19^. HLA class I is then ubiquitinated, dislocated and subsequently directed towards the proteasome for degradation^13^,^17^,^18^,^19^,^20^,^21^.

The US11-mediated degradation of HLA class I has been instrumental in the identification of key components of mammalian ERAD, such as Derlin-1 (refs 15, 17), p97 (ref. 18), VIMP^17^ and SEL1L^22^. However, a complete understanding of the dislocation complex and its modes of action is currently lacking. Most known pathways of ERAD rely on multispanning transmembrane E3 ubiquitin ligases containing a cytosolic RING domain^23^, such as the yeast Hrd1p^24^ and Doa10 (ref. 25), and the mammalian HRD1 (mammalian homologue of yeast Hrd1p)^26^, AMFR/gp78 (ref. 27), TEB4 (mammalian homologue of yeast Doa10)^28^ and TRC8 (ref. 29). The RING domain of the E3 ubiquitin ligase forms a docking site for an E2 ubiquitin-conjugating enzyme, which in turn catalyses polyubiquitination of target substrates^30^. In the context of US11-induced HLA class I degradation, the E3 ubiquitin ligase responsible has remained elusive.

Functional genomics in mammalian cells has been greatly aided by the use of silencing techniques to study loss-of-function phenotypes. Long-term silencing and analysis of non-transfectable cell lines can be achieved by genomic integration of short-hairpin RNAs (shRNAs) through the use of viral vectors for delivery^31^. As with other RNAi-based approaches, the shRNA utility is limited by the low efficacy of many shRNAs, resulting in high false-negative rates, and by off-target effects, leading to high false-positive rates^32^. To overcome these issues, we recently reported on the use of pooled ultracomplex shRNA libraries where each gene is targeted by many different shRNA sequences^33^,^34^. When combined with deep-sequencing-based readouts^33^,^35^, such pooled shRNA library screens allow accurate massive multiplexing in a controlled, identical environment for all cells.

Here, we construct a novel high-complexity shRNA library targeting all known human protein-encoding genes and subsequently perform a genome-wide screen to identify novel proteins that are essential for US11-mediated HLA class I degradation. Besides known players, we identify the previously uncharacterized TMEM129 and the ubiquitin-conjugating E2 enzyme UBE2J2 as essential components for US11-mediated HLA class I downregulation. We demonstrate that TMEM129 is a C4C4-type RING E3 ubiquitin ligase, of which its RING domain is essential for US11-mediated HLA class I downregulation. Co-immunoprecipitation studies indicate that TMEM129 is present in US11-containing dislocation complexes. Besides the previously reported UBE2K, also UBE2J2 is essential for US11-mediated HLA class I downregulation and it is part of TMEM129-containing dislocation complexes. Notably, TMEM129 is associated with various dislocation components independently of US11, suggesting that TMEM129 might be involved in ERAD of proteins outside the context of HCMV US11-mediated HLA class I degradation.

## Results

### An shRNA screen identifies TMEM129 as a player in HLA-I ERAD

We have previously established a screening platform in which pooled shRNA library screens were coupled to deep-sequencing as a means to perform large RNAi screens in a single population of cells^33^,^34^,^36^. Here, we constructed a new pooled lentiviral shRNA library targeting each annotated human protein-coding gene with ~30 independent shRNAs (see Methods section for details). We exploited this new resource to identify proteins that are essential for US11-mediated HLA class I downregulation in human cells. To this end, U937 monocytic cells were generated expressing a chimaeric HLA-A2 molecule that was N-terminally tagged with eGFP and a Myc-epitope (eGFP-Myc-HLA-A2). In this way, relative total HLA class I expression can be measured by assessing the eGFP signal and surface display can be visualized by flow cytometry using a Myc-specific antibody. As expected, introduction of the HCMV US11 protein into these cells induced potent downregulation of the chimaeric HLA-A2 molecule as well as that of endogenously expressed HLA-A3 (Supplementary Fig. 1a).

**Figure 1:**
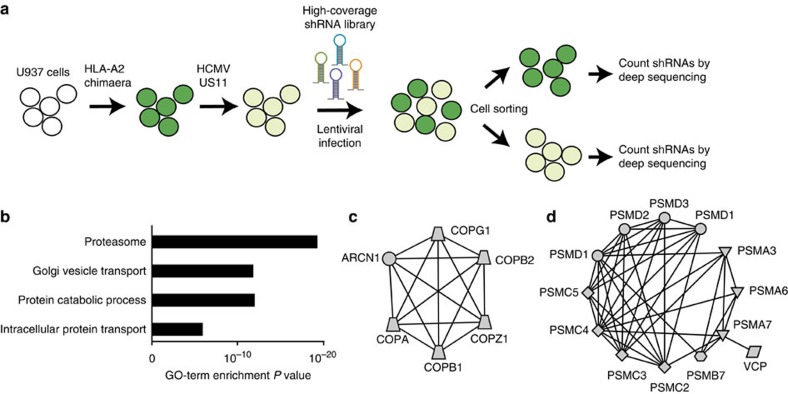
Pooled high-coverage RNAi screen for players involved in US11-mediated ERAD of HLA class I molecules. (**a**) Experimental strategy: U937 cells were transduced with an eGFP-Myc-HLA-A2 chimaera and subsequently transduced with an HCMV US11-expression vector. The resulting cells were cloned and displayed low total eGFP-Myc-HLA-A2 expression as assessed by eGFP expression, and low cell-surface expression of the chimaeric (as assessed by anti-Myc cell-surface stain) and endogenous HLA class I alleles. The cells were infected with the pooled genome-wide high-coverage shRNA library and subjected to flow cytometry sorting at 6 dpi to select for eGFP^bright^ cells and eGFP^dim^ control cells. The frequency of shRNA-encoding constructs in each subpopulation was determined by deep sequencing. (**b**) GO-term enrichment analysis for hits from the screen was assessed using the Database for Annotation, Visualization and Integrated Discovery (DAVID). Based on the frequency in the treated and untreated subpopulations, a hit list was established covering genes that were shared among the top 100 enriched genes from two independent screens (Table 1). (**c**,**d**) Network analysis on the selected genes identified two clusters consisting of genes present in the COPI complex (**c**) and proteasome-associated genes (**d**).

A single population of U937 cells co-expressing eGFP-Myc-HLA-A2 and US11 was infected with the pooled shRNA library and the cells were divided into eGFP-bright and -dim populations by FACS sorting. Subsequently, we assessed which shRNAs were enriched in the eGFP-bright population as compared with the eGFP-dim population and performed statistical analysis on these (Fig. 1a). Two independent screens were executed and the overlap of the top 100 enriched genes from both screens is presented as our primary hit list in Table 1. GO term enrichment analysis of the hits showed a strong enrichment for genes involved in Golgi vesicle transport and proteasome-dependent catabolic processes (Fig. 1b). Indeed, network analysis of the hits revealed two clear clusters comprising most members of the coat protein complex involved in retrograde transport of proteins from the Golgi complex back to the rough ER^37^ (Fig. 1c) and numerous proteasome subunits (Fig. 1d). Since it is known that US11-mediated ERAD of HLA class I relies on ubiquitin-dependent proteasomal degradation of the HLA class I substrate^13^, we expected to identify proteasome subunits in our screen. Additionally, the valosin-containing protein (VCP, commonly referred to as p97) was a clear hit. p97 is an AAA ATPase responsible for extracting and shuttling dislocated substrates from the ER environment towards the proteasome for degradation^18^. Besides these, there were numerous genes involved in ubiquitin biology that were clearly enriched in both screens but which did not meet our strict cutoff requirements; these genes included ubiquitin B (UBB), ubiquitin C (UBC), Ubiquitin-Activating Enzyme E1 (UBA1), the E2-conjugating enzyme UBE2J2, the p97-interacting proteins UFD1L and NPLOC4, as well as multiple additional proteasome subunits.

### TMEM129 is essential for HLA class I dislocation by US11

**Figure 2:**
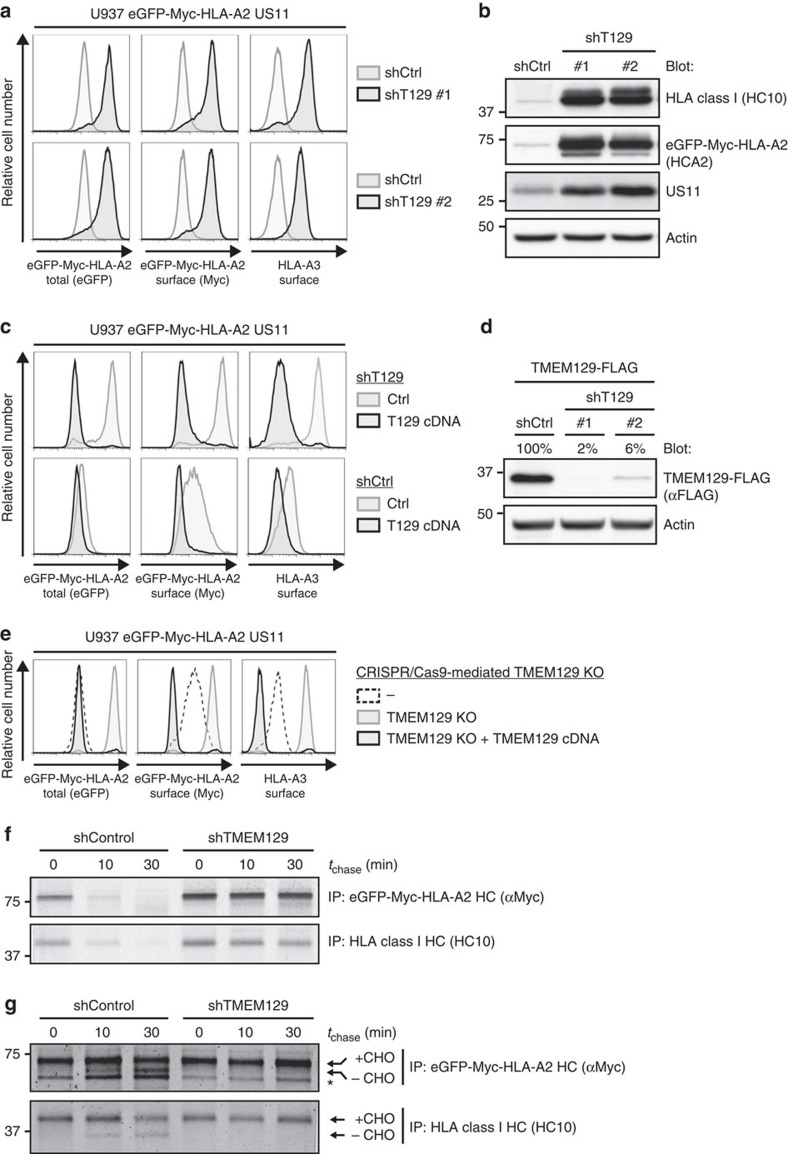
TMEM129 is crucial for US11-mediated HLA class I downregulation. (**a**) Depletion of endogenous TMEM129 by shRNAs induces potent rescue of eGFP-Myc-HLA-A2 and endogenous HLA-A3 in U937 eGFP-Myc-HLA-A2 US11 cells. ShRNAs targeting TMEM129 (black histograms) or control shRNAs (grey histograms) were introduced. Surface endogenous HLA-A3 and surface (Myc) and total (eGFP) eGFP-Myc-HLA-A2 were assessed by flow cytometry (7 dpi). An assessment of TMEM129 levels upon shRNA depletion is presented in Fig. 2d. (**b**) Immunoblot analysis of endogenous HLA class I, eGFP-Myc-HLA-A2, US11, and loading control Actin in mock- (shCtrl) and TMEM129-depleted cells (shTMEM129 no. 1-2). (**c**) Rescue of HLA class I upon TMEM129 depletion is reversed upon overexpression of TMEM129. A TMEM129-expressing vector (black-lined histograms) or an empty vector (grey-lined histograms) were stably introduced in TMEM129- (shT129, upper panels) or mock-depleted (shCtrl, lower panels) cells, after which surface endogenous HLA-A3, and surface (Myc) and total (eGFP) eGFP-Myc-HLA-A2 were assessed by flow cytometry. (**d**) Immunoblot analysis of shRNA-mediated downregulation of overexpressed TMEM129-FLAG. Percentages indicate expression levels compared with mock (shCtrl) depletion normalized against Actin levels. Similar results were obtained for additional TMEM129-targeting shRNAs (Supplementary Fig. 1b–d). Depletion of endogenous TMEM129 by the first shRNA is depicted in Supplementary Fig. 9c). (**e**) CRISPR/Cas-mediated knockout of endogenous TMEM129 induces potent rescue of HLA class I. Surface endogenous HLA-A3, and surface (Myc) plus total (eGFP) expression of eGFP-Myc-HLA-A2 were assessed by flow cytometry in control cells (dashed histogram), TMEM129-null cells (grey-lined histograms) and TMEM129-null cells with reconstituted TMEM129 expression (black histograms). A clonal TMEM129-null cell line was established and stained; additional clones are depicted in Supplementary Fig. 2a,b. (**f**) Mock- (shControl) and TMEM129-depleted U937 eGFP-Myc-HLA-A2 US11 cells were subjected to a pulse chase analysis. Cells were radioactively labelled for 10 min, and chased for the indicated timeframes. Subsequently, HLA class I HCs were immunoprecipitated from lysates using indicated antibodies. (**g**) TMEM129 depletion in the presence of a proteasome inhibitor abrogates dislocation of HLA class I. Similar experiment as in **f**, albeit in the presence of the proteasome inhibitor MG132 (20 μM) immediately before and during the experiment. N-linked glycosylated (+CHO) and deglycosylated (-CHO) HLA class I HCs are indicated. The asterisk indicates a non-specific background band.

We focused our validation analysis on the uncharacterized protein TMEM129. Depletion of TMEM129 by using independent shRNAs induced potent rescue of both the chimaeric HLA-A2 and the endogenous HLA-A3 expression in U937 cells co-expressing US11 (Fig. 2a; Supplementary Fig. 1b). Consistent with the flow cytometry data, immunoblot analysis for HLA class I expression revealed increased protein levels in the presence of TMEM129 shRNAs (Fig. 2b; Supplementary Fig. 1c). Intriguingly, US11 protein levels were upregulated upon TMEM129 depletion as well. The impaired HLA class I downregulation in TMEM129-depleted cells was restored upon overexpression of TMEM129 (Fig. 2c, upper panels), which excludes the possibility that HLA class I rescue was caused by off-targeting effects of the used shRNAs. Notably, overexpression of TMEM129 even enhanced HLA class I downregulation in both TMEM129- and mock-depleted cells (Fig. 2c, lower panels). The efficiency of shRNA-mediated TMEM129 depletion is depicted in Fig. 2d. Similar results were obtained for additional TMEM129-targeting shRNAs (Supplementary Fig. 1b–d).

Besides validating TMEM129 by using RNAi, we employed the recently established CRISPR/Cas genome-engineering system to generate TMEM129-null cells. Expression of a *TMEM129*-targeting guideRNA (gRNA) induced potent rescue of both chimaeric and endogenous HLA class I molecules to levels comparable to those in US11-negative cells (Fig. 2e and Supplementary Fig. 2a,b). Loss of the TMEM129 protein was confirmed by TMEM129 immunoprecipitation experiments (Supplementary Fig. 9d). Subsequent reconstitution of TMEM129 restored US11-mediated HLA class I downregulation in TMEM129-null cells, and even reduced HLA class I protein levels to almost undetectable levels (Fig. 2e and Supplementary Fig. 2b, lower panels).

US11 induces rapid dislocation of HLA class I molecules from the ER to the cytosol, where HLA class I is subsequently degraded by the proteasome (Supplementary Fig. 3)^13^. Upon dislocation into the cytosol, HLA class I HCs are deglycosylated, and these species can be visualized upon proteasome inhibition^13^. We performed pulse chase experiments to determine whether TMEM129 depletion impairs HLA class I HC dislocation and/or its subsequent degradation by the proteasome. As expected, TMEM129 depletion greatly stabilized HLA class I in the presence of US11 (Fig. 2f). Upon proteasome inhibition, we observed accumulation of deglycosylated HCs in control cells, whereas these species were completely absent in cells depleted for TMEM129 (Fig. 2g). Results were comparable for eGFP-Myc-HLA-A2 and endogenous HLA class I HCs (compare upper and lower panels in Fig. 2f,g). These data show that TMEM129 is critically involved in US11-mediated HLA class I dislocation and degradation.

### TMEM129 is a novel E3 ubiquitin ligase

**Figure 3:**
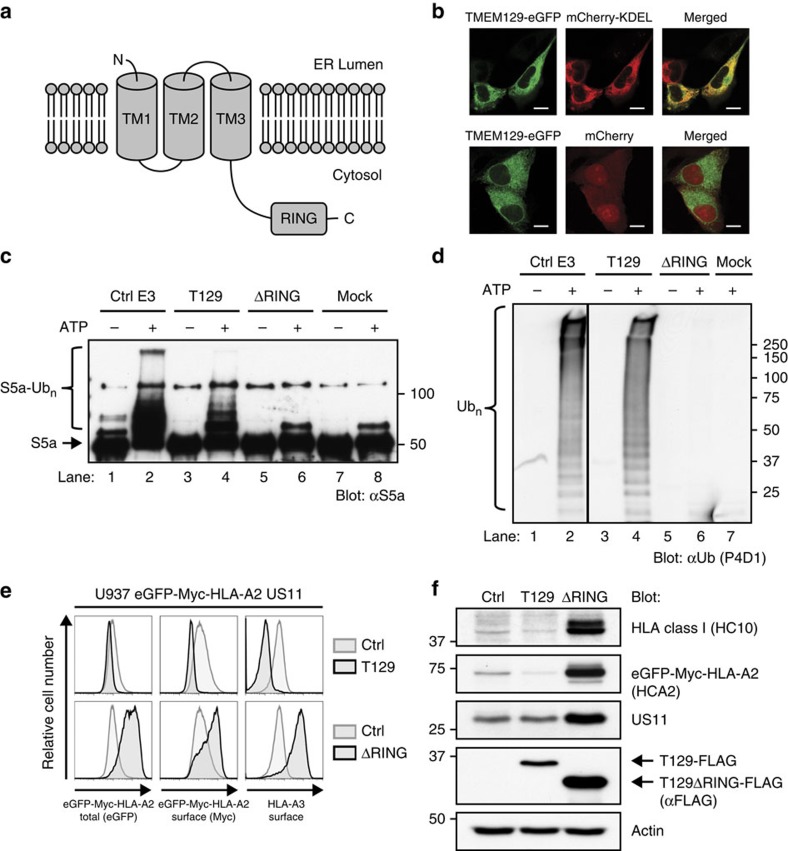
TMEM129 is an E3 ubiquitin ligase essential for US11-mediated HLA class I downregulation. (**a**) Predicted topology of TMEM129 using the TOPCONS prediction server. TM: transmembrane domain; RING: RING domain. The N- and C terminus of the protein are depicted. (**b**) TMEM129 localizes to the ER. MelJuSo cells stably co-expressing TMEM129-eGFP with either mCherry (cytosolic and nuclear) or mCherry-KDEL (ER-localized) were subjected to fluorescent confocal microscopy to assess colocalization of TMEM129-eGFP with either marker. White bars represent 10 μm. (**c**) TMEM129 is an E3 ubiquitin ligase. *In vitro* ubiquitination assays were performed by using purified ubiquitin, E1 enzyme UBA1, E2 enzyme UBE2D3 and E3 enzyme MuRF1 (ctrl E3), immunoprecipitated TMEM129-FLAG-ST2 (T129) or TMEM129ΔRING-FLAG-ST2 (ΔRING), in the absence or presence of ATP. Purified S5a was used as a substrate. Immunoblot analysis was performed using an anti-S5a antibody to visualize poly-ubiquitinated S5a. Addition of the tags did not interfere with TMEM129 function (see Supplementary Fig. 10c). (**d**) The experiment was performed as in Fig. 3c, although a specific substrate was omitted from the reaction. Immunoblot analysis was performed using the anti-ubiquitin P4D1 mAb to visualize polyubiquitin (Ub_*n*_). (**e**) The TMEM129 RING domain is essential for US11-mediated HLA class I downregulation. Flow cytometry analysis of endogenous surface HLA-A3, and surface (Myc) and total (eGFP) eGFP-Myc-HLA-A2 in U937 eGFP-Myc-HLA-A2 cells expressing US11 and co-expressing either TMEM129 (T129), TMEM129ΔRING (ΔRING) or an empty vector (ctrl). (**f**) Same cells as in Fig. 3e, now analysed by immunoblotting for the indicated proteins. TMEM129-FLAG retained its ability to enhance HLA class I downregulation (Supplementary Fig. 10a).

TMEM129 is an uncharacterized transmembrane protein of 362 amino acids that is predicted to have 3 transmembrane domains (TOPCONS; Fig. 3a), and is localized to the ER (Fig. 3b). The protein contains a putative unconventional C4C4 RING domain, suggesting that TMEM129 might be an E3 ubiquitin ligase. To test this, FLAG- and Strep-tag II-tagged versions of TMEM129 and RING-less TMEM129 were immunoprecipitated from U937 cell lysates and used in *in vitro* ubiquitination assays. Incubation of TMEM129 with E1 UBA1, the promiscuous E2 UBE2D3, S5a (Rpn10) protein substrate, ubiquitin and ATP resulted in the formation of poly-ubiquitinated S5a species (Fig. 3c, lane 4), whereas removal of the RING domain completely abrogated this activity (lane 6). Also, in the absence of S5a substrate, TMEM129 induced the formation of polyubiquitin chains (Fig. 3d, lane 4), whereas the RING-less mutant did not (lane 6).

Next, we investigated whether the RING domain of TMEM129 is required for US11-mediated HLA class I downregulation. Expression of the full-length TMEM129 enhanced HLA class I downregulation (Fig. 3e, upper panels), whereas expression of RING-less TMEM129 in U937 eGFP-Myc-HLA-A2 US11 cells resulted in a dominant-negative phenotype, that is, rescue of surface HLA-A3, and total and surface eGFP-Myc-HLA-A2 (Fig. 3e, lower panels). These results were confirmed by immunoblotting experiments (Fig. 3f), in which increased levels of HLA class I HCs were observed in the presence of RING-less TMEM129. Again, increased US11 protein levels were observed, this time upon RING deletion. The RING-less TMEM129 data recapitulate our findings obtained by TMEM129 depletion via shRNAs and CRISPR gRNAs. In conclusion, these findings identify TMEM129 as an E3 ubiquitin ligase with a C4C4 RING domain, removal of which results in a dominant-negative phenotype rescuing HLA class I from US11-mediated dislocation.

### TMEM129 is part of the US11 dislocation complex

**Figure 4:**
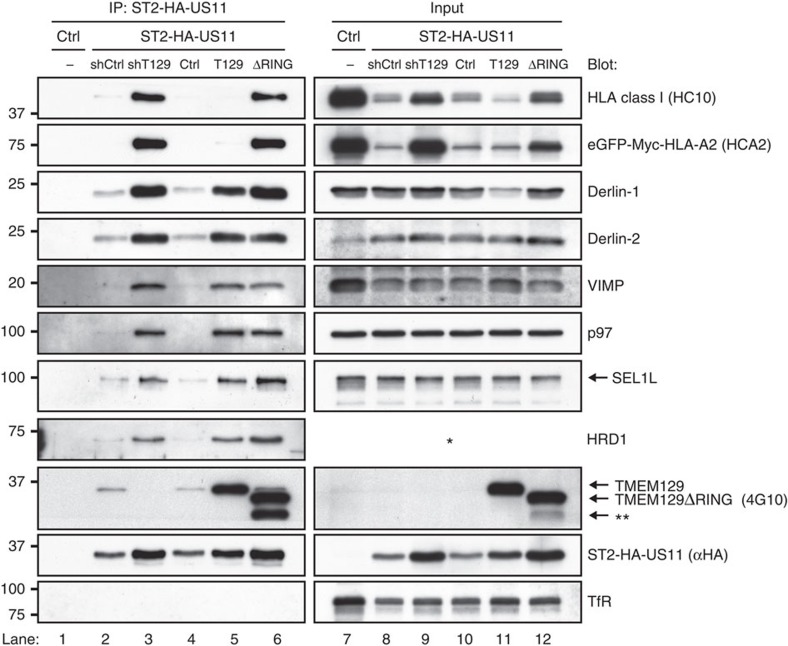
TMEM129 is part of the US11 dislocation complex. Strep-tag II- and HA-tagged US11 was immunoprecipitated using StrepTactin beads from 1.0% digitonin lysates of US11-negative (lane 1) and positive (lanes 2–6) U937 eGFP-Myc-HLA-A2 cells. The US11-expressing cells were co-expressing a control shRNA (shCtrl, lane 2), an shRNA targeting TMEM129 (shT129, lane 3), an empty vector (ctrl, lane 4), TMEM129 (T129, lane 5) or TMEM129ΔRING (ΔRING, lane 6). Immunoprecipitated proteins were eluted using d-Desthiobiotin, after which immunoblot analysis was performed for proteins indicated. The right panels (lanes 7–12) indicate loading controls to analyse input of the indicated proteins prior immunoprecipitation. The Strep-tag II- and HA-tagged US11 retained its ability to downregulate HLA class I molecules (Supplementary Fig. 10b). The HRD1 antibody could not detect HRD1 in digitonin cell lysates, indicated by the asterisk. The double asterisk indicates an additional unspecified truncated form of TMEM129ΔRING.

To assess whether TMEM129 is present in the US11-containing dislocation complex, we generated N-terminally Strep-tag II and HA-tagged US11 molecules (ST2-HA-US11) and subjected these to co-immunoprecipitation experiments. Endogenous TMEM129 co-precipitated with ST2-HA-US11 in control cells (Fig. 4, lanes 2 and 4), but not in TMEM129-depleted cells (lane 3). High levels of overexpressed TMEM129 and TMEM129ΔRING (lanes 5 and 6) were co-precipitated with US11, together with Derlin-1, Derlin-2, VIMP, p97, SEL1L and HRD1. Depletion of TMEM129 using shRNAs (lane 3) or removal of the RING domain (lane 6) did not abrogate association of Derlin-1, Derlin-2, VIMP, p97, SEL1L and HRD1. Notably, HLA class I HCs were exclusively detectable in complexes isolated from TMEM129-depleted (lane 3) or TMEM129ΔRING-expressing (lane 6) cells. Taken together, our data show that TMEM129 is part of the US11-containing dislocation complex.

### UBE2J2 is essential for US11-mediated HLA class I ERAD

**Figure 5:**
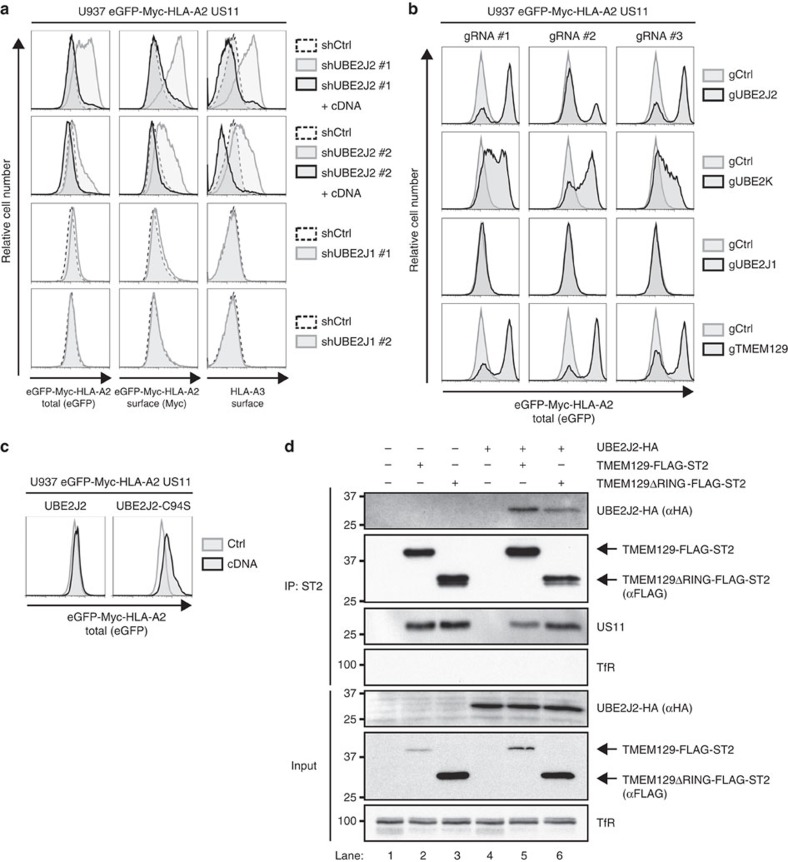
UBE2J2 is essential for US11-mediated HLA class I downregulation. (**a**) UBE2J2 depletion by shRNAs induces rescue of HLA class I in US11-expressing cells. Two individual UBE2J2- and UBE2J1-targeting shRNAs (grey-lined histograms), two individual UBE2J2-targeting shRNAs together with UBE2J2 cDNA (black-lined histograms), or one control shRNA (dashed histograms) were introduced in U937 eGFP-Myc-HLA-A2 US11 cells. The flow cytometry analysis of endogenous surface HLA-A3, and surface (Myc) and total (eGFP) eGFP-Myc-HLA-A2 expression was performed at 7 dpi. (**b**) CRISPR/Cas-mediated knockout of UBE2J2 and UBE2K induce potent rescue of HLA class I. Total (eGFP) expression of eGFP-Myc-HLA-A2 were assessed by flow cytometry in U937 eGFP-Myc-HLA-A2 US11 control cells (grey histogram) and cells knocked out for either UBE2J2 (black histograms, upper panels), UBE2K (black histograms; second panels), UBE2J1 (black histograms, third panels) or TMEM129 (black histograms, lower panels) using three individual CRISPR gRNAs. (**c**) Dominant-negative UBE2J2 (C94S) causes rescue of HLA class I. Total (eGFP) expression of eGFP-Myc-HLA-A2 was assessed by flow cytometry in U937 eGFP-Myc-HLA-A2 US11 control cells (grey histogram) and cells expressing either wild-type UBE2J2 (black histogram, left panel) or dominant-negative UBE2J2-C94S (black histogram, right panel). (**d**) UBE2J2 associates with TMEM129. Strep-tag II- and FLAG-tagged TMEM129 was immunoprecipitated using StrepTactin beads from 1.0% digitonin lysates of U937 eGFP-Myc-HLA-A2 US11 cells expressing indicated constructs. The Strep-tag II- and FLAG-tagged TMEM129 retained its ability to enhance HLA class I downregulation (Supplementary Fig. 10c, left panels). Immunoprecipitated proteins were eluted using d-Desthiobiotin, after which immunoblot analysis was performed for the proteins indicated.

Ubiquitination of a target substrate is often facilitated by more than one E2 ubiquitin-conjugating enzyme^38^. Currently, the identity of one E2 ubiquitin-conjugating enzyme contributing to US11-mediated HLA class I downregulation is known, namely UBE2K/E2-25K (ref. 39). The combined results of our two genome-wide shRNA screens did not identify any additional candidates (Table 1). However, we did observe a clear enrichment for the E2 UBE2J2. We therefore tested UBE2J2-targeting shRNAs, which indeed rescued endogenous and chimaeric HLA class I molecules in US11-expressing cells (Fig. 5a, upper two panels). This rescue was completely abolished upon co-expression of an UBE2J2 cDNA, showing that the observed phenotype was caused by on-target effects of the UBE2J2-targeting shRNAs. These results were confirmed by the generation of UBE2J2-null cells using the CRISPR/Cas system (Fig. 5b, upper panel). ShRNAs (Fig. 5a, lower two panels) and CRISPR gRNAs (Fig. 5b, third row) targeting the homologous UBE2J1 did not have any effect on HLA class I expression. Expression of a dominant-negative UBE2J2 (C94S) increased HLA class I expression in US11-expressing cells (Fig. 5c). Co-immunoprecipitation experiments showed that UBE2J2 is in the same complex as the E3 ubiquitin ligase TMEM129 (Fig. 5d). RING removal of TMEM129 does not affect association of UBE2J2 with the complex. Intriguingly, UBE2K-null cells showed a clear rescue of HLA class I molecules (Fig. 5b, second row), suggesting that both UBE2J2 and UBE2K are essential for HLA class I downregulation by US11.

### TMEM129 is an integral component of dislocation complexes

**Figure 6:**
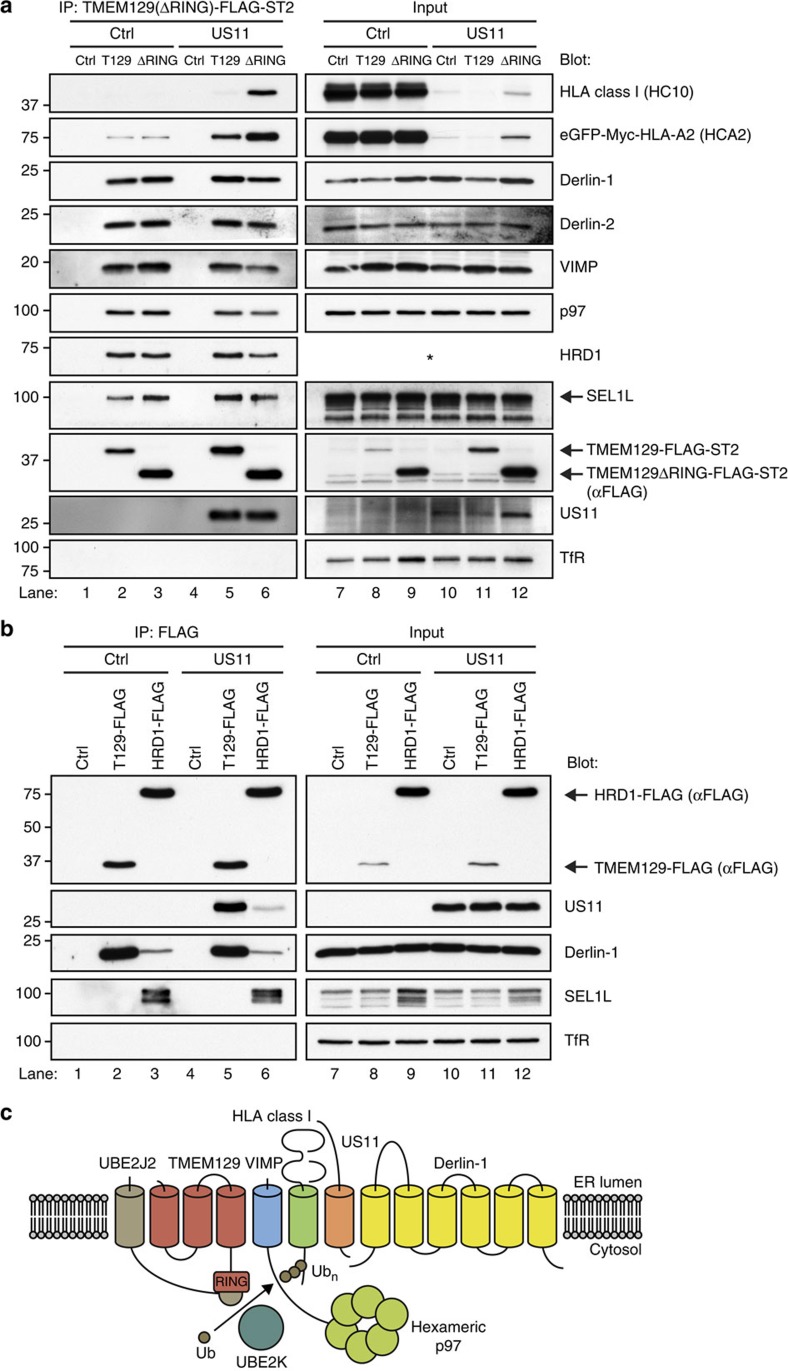
TMEM129 is present in ERAD complexes in the absence of US11. (**a**) C-terminally Strep-tag-II- and FLAG-tagged TMEM129 or TMEM129ΔRING were immunoprecipitated using StrepTactin beads from 1.0% digitonin lysates of US11-negative (lanes 1–3) and US11-positive (lanes 4–6) U937 eGFP-Myc-HLA-A2 cells. The cells were co-expressing a control vector (ctrl, lanes 1 and 4), TMEM129-FLAG-ST2 (T129, lanes 2 and 5), or TMEM129ΔRING-FLAG-ST2 (ΔRING, lanes 3 and 6). Immunoprecipitated proteins were eluted using d-Desthiobiotin, after which immunoblot analysis was performed for the proteins indicated. The right panels (lanes 7–12) indicate loading controls to analyse input of the indicated proteins before immunoprecipitation. The C-terminally Strep-tag-II- and FLAG-tagged TMEM129 construct retained its ability to enhance US11-mediated HLA class I downregulation (Supplementary Fig. 10c, left panels), whereas the C-terminally tagged TMEM129ΔRING retained its dominant-negative phenotype (Supplementary Fig. 10c, right panels). The HRD1 antibody could not detect HRD1 in digitonin cell lysates, indicated by the asterisk. (**b**) C-terminally FLAG-tagged TMEM129 and HRD1 were immunoprecipitated using FLAG-M2-coupled beads from 1.0% digitonin lysates of US11-negative (lanes 1–3) and US11-positive (lanes 4–6) U937 eGFP-Myc-HLA-A2 cells. Immunoprecipitated proteins were eluted using FLAG peptides, after which immunoblot analysis was performed for the proteins indicated. (**c**) Schematic overview of the US11 dislocation complex. The depicted location of the proteins is not accurate.

We next assessed whether TMEM129 is part of ER-resident dislocation complexes independent of US11, which might indicate that TMEM129 plays a role in general ERAD. Co-immunoprecipitation experiments were performed using C-terminal Strep-tag II- and FLAG-tagged TMEM129 and TMEM129ΔRING. Both proteins were found to be associated with Derlin-1, Derlin-2, VIMP, p97, SEL1L and HRD1 to similar levels in both US11-expressing and US11-negative cells (Fig. 6a). In the absence of US11, no or very low levels of HLA class I and eGFP-Myc-HLA-A2 associated with TMEM129, despite the presence of considerable amounts of HLA class I molecules in these cells (compare upper two panels, lanes 2 and 8, and lanes 3 and 9). In the presence of US11, again increased levels of HLA class I were found in association with TMEM129ΔRING as compared with wild-type TMEM129 (compare lanes 5 and 6, upper two panels).

As HRD1 and SEL1L co-precipitated together with US11 (Fig. 4) and TMEM129 (Fig. 6a), we compared overexpressed TMEM129 and HRD1 in their ability to associate with US11, Derlin-1 and SEL1L (Fig. 6b). Immunoprecipitation of TMEM129 recovered higher quantities of US11 and Derlin-1 compared with immunoprecipitation of HRD1-FLAG (compare Fig. 6b, lanes 5 and 6). In addition, immunoprecipitation of HRD1 recovered SEL1L (lane 6), which was not observed upon TMEM129 pull-down (lane 5). The same results were observed when US11 was absent (lanes 1–3). Thus, TMEM129, and not HRD1, preferentially associates with US11 and Derlin-1. Moreover, SEL1L preferentially interacts with HRD1 and not with TMEM129. These results demonstrate that TMEM129 is an integral constituent of the ER-resident dislocation complexes, suggesting that this E3 ubiquitin ligase might play a role in physiological ERAD, independently of US11. A model of the US11 dislocation complex is depicted in Fig. 6c.

## Discussion

HCMV US11-mediated degradation of HLA class I molecules serves as a paradigm for ERAD and has facilitated the identification of multiple essential components of this important degradation pathway. In this study, we identified additional essential players of US11-mediated HLA class I downregulation by performing a pooled genome-wide shRNA library screen in human cells expressing the US11 protein. To this end, we constructed a new high-complexity genome-wide shRNA library consisting of ~550,000 different shRNAs. We assessed whether we could successfully conduct a genome-wide screen using the entire library in a single population of cells. This approach proved successful, as we identified known players, such as p97 and a large compendium of proteasome subunits. Besides this, we identified multiple new genes potentially involved, such as subunits of the COPI retrograde trafficking machinery and the uncharacterized TMEM129. We chose a strict cutoff for our validation experiments and only allowed the overlap of the top 100 enriched genes from two independent screens in our hit list. This screen is the first to show that ultracomplex libraries with over 550,000 different shRNAs can be successfully applied in genetic screens in a single population of cells using deep-sequencing as a read-out for library complexity and dynamics.

Our hit list contained multiple genes of the COPI retrograde transport system. In agreement with this, we observed a moderate rescue of eGFP-Myc-HLA-A2 expression in COPI-depleted cells co-expressing the chimaeric molecule and US11 (Supplementary Fig. 4a). Since immature HLA class I molecules that escape to the Golgi complex are efficiently recruited back to the ER in a COPI-dependent manner^40^, depletion of COPI subunits would theoretically cause accumulation of these HCs in the Golgi, which could protect these from US11-induced ERAD initiated in the ER. Indeed, we observed accumulation of both endogenous and chimaeric HLA class I molecules in a post-ER compartment upon COPI depletion, as EndoH-resistant HCs became apparent in immunoblot analysis (Supplementary Fig. 4b). Additionally, confocal studies clearly demonstrated a relocalization of chimaeric HLA class I molecules to the Golgi upon COPI depletion (Supplementary Fig. 4c). Rescue of HLA class I in COPI-depleted cells could be related to the inability of ER-localized US11 to initiate degradation of Golgi-localized HCs, or by a lack of ERAD components in the ER due to an impaired retrieval of membrane proteins from the Golgi to the ER upon blockage of the retrograde transport system. We therefore believe that the COPI complex is not specifically involved in US11-mediated HLA class I ERAD, but that the identification of these subunits in the screen is likely caused by an indirect effect.

Our hit list contained the previously uncharacterized protein TMEM129. Subsequent validation experiments, including shRNA-mediated knockdown, CRISPR/Cas-mediated knockout and overexpression of TMEM129, showed that TMEM129 is crucial for US11-mediated HLA class I downregulation. Sequence analysis revealed that TMEM129 contains a putative non-classical RING domain. Whereas a conventional RING domain contains three zinc-coordinating cysteine pairs and one cysteine-histidine pair, the RING domain of TMEM129 possesses four zinc-coordinating cysteine pairs (C4C4) that are fully conserved among a wide range of TMEM129 orthologs (Supplementary Fig. 5). Thus far, only a single mammalian E3 ubiquitin ligase (CNOT4) has been described to contain a C4C4 RING domain^41^. We show that TMEM129 is a *bona fide* E3 ubiquitin ligase as it possesses such activity *in vitro*, and removal of the RING domain completely abrogated this activity. In line with this, expression of RING-less TMEM129 in US11-expressing cells caused a dominant-negative phenotype of increased HLA class I levels. Not only HLA class I levels were found to be elevated but also US11 protein levels were increased upon expression of RING-less TMEM129, an observation also made upon shRNA-mediated depletion of TMEM129. However, when TMEM129 was overexpressed in US11-expressing cells, US11 levels remained unchanged, whereas HLA class I levels decreased to almost undetectable levels. Thus, while we cannot rule out that US11 is a substrate for TMEM129 to some extent, we show that TMEM129 primarily mediates dislocation of HLA class I molecules. Immunoprecipitation of US11 and TMEM129 demonstrated that TMEM129 is an integral part of the US11 dislocation complex. Depletion of TMEM129 and expression of RING-less TMEM129 appear to lock the dislocation complex in a dislocation-incompetent state, indicated by high levels of HLA class I in complex with US11, while normally, association of HLA class I with US11 is transient^15^. The presence of TMEM129 in ERAD complexes independently of US11 suggests that the protein may also be involved in dislocation of other cellular substrates. Among these substrates may be cellular proteins destined for degradation in the context of ER quality control and/or alleviation of ER stress through the unfolded protein response (UPR). CRISPR/Cas-mediated TMEM129 knockout results in a slight induction of the UPR as assessed by XBP-1 splicing analysis (Supplementary Fig. 6), suggesting that TMEM129 might play a role in this process.

The identification of the E3 ubiquitin ligase crucial for US11-mediated HLA class I downregulation has been a subject of intense investigation. Currently, only a few E3 ubiquitin ligases involved in human ERAD are known, of which HRD1 is the most studied. However, previous attempts to link HRD1 to US11-mediated HLA class I degradation have not been successful, as HRD1 depletion studies did not rescue HLA class I from US11-mediated degradation^42^. In line with this, we did not observe increased US11-mediated HLA class I downregulation upon HRD1 overexpression (Supplementary Fig. 7a), nor did we see rescue of HLA class I from US11-mediated degradation upon either shRNA-mediated HRD1 depletion or gRNA-mediated *HRD1* gene disruption (Supplementary Fig. 7c,e respectively). Nevertheless, HRD1 was present in US11 and TMEM129-containing dislocation complexes (Figs 4 and 6a). A subsequent immunoprecipitation experiment to compare the binding of HRD1 and TMEM129 to other dislocation components revealed that US11 and Derlin-1 associated more strongly with TMEM129, compared with HRD1 (Fig. 6b). In this context, SEL1L was preferentially associated with HRD1, and not with TMEM129, even though both E3 ligases were immunoprecipitated to similar quantities. Hence, SEL1L may thus be recruited to the dislocation complex through its association with HRD1, which is also present in the complex, albeit in small amounts. We therefore propose that TMEM129 is the E3 ubiquitin ligase that is essential for US11-mediated HLA class I degradation.

The fact that TMEM129 has escaped identification thus far^43^ is likely due to its low endogenous expression levels, which complicates its identification via standard biochemical approaches. Additionally, TMEM129 was a previously uncharacterized protein, which prevented including TMEM129 as a candidate in focused E3 ubiquitin ligase screening approaches by, for example RNAi. Our strategy involved the use of an unbiased genome-wide shRNA library screen in which all protein-encoding genes were included, thereby eliminating these hurdles and illustrating the advantages of genome-wide pooled shRNA library screens.

E3 ubiquitin ligases collaborate with E2 enzymes to facilitate polyubiquitination of a target substrate^30^. Previously, the E2 UBE2J1 was implicated to be involved in US11-mediated HLA class I dislocation^43^. Unexpectedly, not only the dominant-negative UBE2J1 but also the wild-type E2 protein was found to impair US11-mediated HLA class I dislocation^43^. We now show that UBE2J2, and not UBE2J1, is essential for HLA class I degradation by US11, since depletion or knockout of UBE2J2, but not UBE2J1, causes a dramatic rescue of HLA class I from US11-mediated degradation. Considering that mammalian UBE2J1 and UBE2J2 are highly homologous^44^, UBE2J1 might compete with UBE2J2 for binding to certain E3 ubiquitin ligases; when present in excess, UBE2J1 might prevent UBE2J2 from binding, which could explain the earlier observations. Additionally, co-immunoprecipitation experiments confirmed that UBE2J2 is present in complexes with TMEM129. Also, the E2 UBE2K/E2-25K has been reported to facilitate ubiquitination of HLA class I in permeabilized US11-expressing cells^39^. In this experimental setup however, membrane-anchored E2 enzymes (for example, UBE2J2) remained uninvestigated since the usurped semi-permeabilized system only allowed removal and addition of cytosolic E2 enzymes. Using CRISPR/Cas-mediated gene disruption, we show that UBE2K is also essential for US11-mediated HLA class I downregulation, thereby confirming the previous findings for UBE2K. UBE2J2- and UBE2K-null cells expressing US11 both show high levels of rescued HLA class I, which indicates that these two E2 enzymes are not interchangeable and catalyse two different ubiquitination steps essential for the dislocation process.

The processivity of polyubiquitin formation is often orchestrated by at least two different E2 enzymes, one of which is responsible for ubiquitin chain initiation, while the other E2 promotes ubiquitin chain elongation^38^. Mono-ubiquitination of HLA class I is not sufficient to induce US11-mediated dislocation; completion of this reaction requires Lys48-linked polyubiquitination^21^,^45^. While UBE2K is known to catalyse Lys48-linked ubiquitin chain elongation, and not chain initiation^46^, it is unknown what type of ubiquitin chain formation is catalysed by UBE2J2. However, in the context of US11-mediated HLA class I degradation, the membrane-bound UBE2J2 might promote mono-ubiquitination of HLA class I, thereby enabling the cytosolic UBE2K to catalyse Lys48-linked ubiquitin chain elongation. Removal of either UBE2J2 or UBE2K would prevent polyubiquitination of HLA class I and thereby inhibit its dislocation. This hypothesis would also be in line with the results previously obtained for UBE2K using the semi-permeabilized cells^39^; in this system, the membrane-bound UBE2J2, which was not removed, would enable the supplied UBE2K to catalyse ubiquitin chain elongation.

Besides US11, the HCMV-encoded protein US2 also mediates dislocation of HLA class I^48^. Whereas US11 uses the E3 ubiquitin ligase TMEM129, US2 relies on the activity of TRC8 (ref. 29). The ERAD pathway exploited by US2 differs from US11-mediated HLA class I degradation in several other ways, including the lack of Derlin-1 involvement. When the degradation of HLA class I HCs is accelerated via depletion of β_2_m, yet another E3 ubiquitin ligase, HRD1, catalyses the degradation of HLA class I HCs^42^,^47^. It is notable that the dislocation of a single degradation substrate may be catalysed by at least three different E3 ubiquitin ligases, depending on its context. This observation testifies to the versatility of ER-associated protein degradation and underscores the essential role that E3 ubiquitin ligases play in regulating the dislocation of substrates into the cytosol for degradation by the ubiquitin–proteasome system.

## Methods

### Cell culture and lentiviral infection

U937 human monocytic cells, 293T human embryonic kidney cells and MelJuSo (MJS) human melanoma cells were obtained from ATCC (American Type Culture Collection) and grown in RPMI medium supplemented with glutamine, penicillin/streptomycin and 10% FCS. For individual gene infections using lentiviruses, virus was produced in 24-well plates using standard lentiviral production protocols, third-generation packaging vectors, and 50 ul viral supernatant adjusted to 8 mg ml^−1^ polybrene was used to infect ~20,000 cells by spin infection at 1,000*g* for 2 h at 33 °C.

### Plasmids

Several different lentiviral vectors were used in the present studies. The N-terminally eGFP and Myc-tagged human HLA-A2 vector present in the lentiviral pHRSincPPT-SGW vector was kindly provided by Dr Paul Lehner and Dr Louise Boyle (University of Cambridge, Cambridge, UK). The pooled shRNA library vector is described in the ‘Genome-wide shRNA screen’ section in the Methods section. Individual shRNAs were derived from the Mission shRNA library (Sigma-Aldrich, St Louis, MO, USA). Typically, we tested five different shRNAs per gene. The ones that were used for figures in this manuscript are presented in Supplementary Table 1. For rescue and overexpression experiments, we cloned tagged and untagged cDNAs from TMEM129, UBE2J1, UBE2J2, HRD1 and US11 in a dual promoter lentiviral vector (no. 2025.pCCLsin.PPT.pA.CTE.4x-scrT.eGFP.mCMV.hPGK.NGFR.pre, kindly provided by Dr Luigi Naldini, San Raffaele Scientific Institute, Milan, Italy). This vector was altered to: replace the minimal CMV promoter with the human EF1A promoter to facilitate potent expression in immune cells; replace the eGFP with a cassette containing several unique restriction sites facilitating DNA cloning (NheI, PacI, PmeI, AfeI, SphI, SphI and NsiI); and replace the NGFR gene with various combinations of fluorescent proteins (mCherry, mAmetrine) and selection markers (PuroR, BlastR, ZeoR and HygroR), which were fused together by means of the ribosomal skipping peptide T2A. The choice of vector used was dictated by the presence of other fluorescent and selection markers in the cell lines in question. For confocal colocalization studies, we used the same dual promoter lentiviral vectors described above in which we either cloned TMEM129 C-terminally fused to a FLAG-epitope and eGFP (TMEM129-eGFP), mCherry alone (mCherry), mCherry N-terminally tagged to amino acids 1–60 of the human galactosyltransferase (GalT-mCherry), or mCherry fused to a C-terminal BiP-leader and N-terminal KDEL-sequence (mCherry-KDEL).

RING-less TMEM129 was generated by removing amino acids 285–362. Dominant-negative UBE2J2 was generated by mutating the cysteine of the active site at position 94 into a serine, as described previously^44^. When indicated, we used the following (epitope) tags: HA-tag (YPYDVPDYA), Myc-tag (EQKLISEEDL), FLAG-tag (DYKDDDDK) and triple Strep-Tag II (ST2; WSHPQFEKGSWSHPQFEKGSWSHPQFEKGS). For CRISPR/Cas experiments, we obtained vectors from the Church lab^49^ via Addgene (Addgene plasmid 41824: gRNA_Cloning Vector, and Addgene plasmid 41815: Cas9 expression vector). Additionally, we constructed a selectable lentiviral CRISPR/Cas vector (which will be described elsewhere, manuscript in preparation) to facilitate efficient and selectable expression of Cas9 and gRNAs in target cells: briefly, the lentiviral pSicoR vector (Jacks Lab, MIT) was altered to express a nuclear-localized Cas9 gene that was N-terminally fused to puroR and a T2A sequence. Additionally, the region immediately downstream of the U6 promoter was replaced by a cassette consisting of two unique restriction sites (BsmBI) to allow cloning of gRNA target sites followed by the gRNA scaffold and a terminator consisting of 5 T-residues.

### Pooled shRNA libraries

We designed shRNAs targeting all protein-encoding transcripts in human cells using the shRNA retriever programme^50^, which generates shRNAs with 22-nucleotide guide strands. We designed oligos consisting of the shRNA hairpin (fully complementary 22-bp arms, and a 9-bp loop) surrounded by 3′ and 5′ primer-binding sites. The shRNAs include an initial G before the sense arm, for correct expression by the U6 promoter, a complementary C after the antisense arm, and a TTTTT (UUUUU in the RNA sequence) terminator to end transcription immediately after the hairpin. Oligonucleotides encoding shRNAs in a sub-library of 55,000 sequences were synthesized by Agilent, and used to generate shRNA libraries as previously described^33^. Twelve sub-libraries were generated where genes were organized into functionally related groups, using GO annotation, curated localization and data from various proteomic surveys of organelles. ShRNAs were cloned into our vector MP-177, for which a detailed map will be provided on request. This vector was derived from the lentiviral pSicoR vector (Jacks Lab, MIT) in which the U6 promoter was altered to allow for sticky cloning of shRNAs in between a BstXI and XhoI site, and in which the CMV promoter was used to drive expression a cassette encoding a puromycin resistance marker, the ribosomal skipping peptide T2A and mCherry. Library quality was evaluated by sequencing 96 colonies from each sub-library, the percentage of perfect sequences ranged from 58 to 76% with an average of 66%. The coverage of each library ranged from 31- to 128-fold with an average of 50-fold, as assessed by bacterial colony counts.

### Genome-wide shRNA screen

U937 monocytic cells co-expressing eGFP-Myc-HLA-A2 and US11 were grown in RPMI medium supplemented with glutamine, penicillin–streptomycin and 10% FCS. Virus was produced in 15-cm plates of 293T cells using standard lentivirus production protocols. For each screen, ~160 × 10^6^ cells were infected with the entire genome-wide shRNA library in 320 ml virus supernatant supplemented with 8 mg ml^−1^ polybrene, divided into wells of a six-well plate and spin-infected at 1,000*g* for 2 h at 33 °C to reach a target infection of ~80%. Six days after infection, ~800 × 10^6^ cells were harvested and subjected to cell sorting via a two-step sort-protocol using a Becton Dickinson Influx cell sorter. First, eGFP-upregulated cells were sorted using an ‘enrichment-protocol’, which allowed for high-speed cell sorting of the entire population of cells in a short timeframe. Next, the selected cells were sorted for purity selecting mCherry-positive cells (cells transduced with an shRNA lentivector) and eGFP-bright cells. The top 0.75% of eGFP^bright^ cells was selected, which resulted in a total of ~2.8 × 10^6^ cells per screen. As control, ~115 × 10^6^ mCherry-positive, eGFP^dim^ cells were sorted. Subsequently, genomic DNA was isolated from the selected cells using standard phenol–chloroform extractions. Next, the lentiviral shRNA inserts were PCR-amplified for 23 cycles using primers AATGATACGGCGACCACCGACACTCTTTCCCacaaaaggaaactcaccctaac and CAAGCAGAAGACGGCATACGAgcggtaatacggttatccacg and Phusion polymerase (NEB) in the presence of buffer GC supplemented with DMSO. These primers contain Illumina adapter sequences (displayed in capital letters) that allow direct loading on an Illumina HiSeq2000 sequencer. As input we performed a PCR on genomic DNA equivalent to ~2.8 × 10^6^ cells for the selected eGFP^bright^ cells, and 20 × 10^6^ cells for the eGFP^dim^ control cells. This resulted in ~35 and 250 50-ul PCR reactions for the selected and control cells, respectively. The PCR products were pooled and purified/concentrated using a PCR purification kit (Qiagen), and subsequently loaded on a 20% polyacrylamide gel in 0.5 × TBE. Bands of the correct size were excised, electro-eluted, purified by phenol-chloroform extraction and subsequently quantified using a Nanodrop quantification device (Nanodrop, Rockland, DE, USA) and an Agilent bioanalyzer (Agilent Technologies, Palo Alto, CA, USA). Deep sequencing was carried out on an Illumina HiSeq2000 (performed by BGI, Hong Kong) using the sequencing primer 5′-GAGACTATAAGTATCCCTTGGAGAACCACCTTGTTGG-3′, in which the guide strands of the shRNA-encoding constructs were detected. Sequences were aligned to the known library sequences using Bowtie^51^ and the counts per shRNA were calculated. On average we obtained ~78 × 10^6^ aligned sequences per sample, which ranged from 56 × 10^6^ cells to 106 × 10^6^ reads per sample. To identify hit genes in the genome-wide screen, we calculated a *P*-value for each gene in our library using a Mann–Whitney *U*-test as described previously^34^. The genome-wide screen was carried out in two independent replicates and the overlap between the top 100 genes with the lowest *P*-values from both screens was selected as hit list. These hits were assessed for enrichment of GO terms using DAVID^52^,^53^ and network analysis was performed using Ingenuity pathway analysis (Ingenuity Systems, Redwood City, CA, USA).

### Generation of CRISPR/Cas-mediated knockout cells

U937 cells co-expressing eGFP-Myc-HLA-A2 and US11 were transfected twice on subsequent days using the Neon Transfection System (Life technologies, Breda, The Netherlands) with a Cas9-encoding vector and a gRNA expression vector targeting human TMEM129. Typically, we obtained ~10% knockout cells as determined by flow-cytometry assessment of upregulated total eGFP-Myc-HLA-A2 (eGFP) levels. Cells were sorted by flow cytometry (FACSAriaII, BD Biosciences) and single-cell cloned by limiting dilution.

Later on, we moved to a lentiviral CRISPR/Cas system (see ‘Plasmids’ section) in which a single lentiviral vector co-expresses a Cas9, puroR and a gRNA sequence. Virus was produced and cells were infected as described in the ‘Cell Culture and Lentiviral Infection’ section. Two days post-infection (dpi), infected cells were selected by using 2 μg ml^−1^ puromycin and allowed to recover. Typically, we observed 30–90% of selected cells to display a full knockout phenotype as assessed by flow cytometry. See also Supplementary Table 2 for gRNA sequences used in this study and Supplementary Fig. 8 for a visualization of the genomic target sites of these gRNAs.

### Antibodies

Primary antibodies used in our studies were mouse α-HLA class I HC HC10 mAb; mouse α-HLA class I HC HCA2 mAb; human α-HLA-A3 OK2F3 mAb (kindly provided by Dr Arend Mulder and Dr Frans Claas (LUMC), 1:40); rabbit α-VIMP pAb (1:500, kindly provided by Y. Ye, NIH, Bethesda, MD, USA)^17^; rabbit α-US11 pAb (1:1,000)^20^; rabbit α-Derlin-1 pAb (no. PM018, MBL, 1:1,000); rabbit α-Derlin-2 pAb (no. PM019, MBL, 1:1,000); mouse α-VCP/p97 18/VCP mAb (no. 612183, BD Transduction Laboratories, 1:1,000); rabbit α-SEL1L pAb (no. S3699, Sigma-Aldrich, 1/1,000), rabbit α-HRD1 pAb (no. AP2184a, Abgent, 1:1,000), mouse α-Ub P4D1 mAb (no. SC-8017, Santa Cruz Biotechnology, 1:400); mouse α-Actin C4 mAb (no. MAB1501R, Millipore, 1:10,000); mouse α-TfR H68.4 mAb (no. 13-68xx, Invitrogen, 1:2,000); mouse α-FLAG-M2 mAb (no. F1804, Sigma-Aldrich, 1:50,000); rat α-HA 3F10 mAb (no. 11867423001, Roche, 1:1,000); mouse α-Myc 9E10 mAb (UCSF hybridoma core, 1:200); mouse α-Myc 9E10-biotin mAb (UCSF hybridoma core, 1:200); rat α-TMEM129 4G10 and 13E2 mAbs (E. Kremmer, Helmholtz Zentrum München, 1:5); mouse α-TMEM129 8D7 and 8G9 mAbs (E. Kremmer, Helmholtz Zentrum München, 1:5).

Secondary antibodies used in our studies were: F(ab')2 fragment goat α-human IgG+IgM(H+L)-PE (no. 109-116-127, Jackson ImmunoResearch, 1:160); streptavidin-BV421 (no. 405226, BioLegend, 1:160); goat α-mouse IgG(H+L)-HRP (no. 170-6516, Bio-Rad, 1:10,000); goat α-rabbit IgG(H+L)-HRP (no. 4030-05, Southern Biotech, 1:10,000); mouse α-rabbit IgG(L)-HRP (no. 211-032-171, Jackson Immunoresearch, 1:10,000); goat α-mouse IgG(L)-HRP (no. 115-035-174, Jackson Immunoresearch, 1:10,000); Goat α-rat IgG(L)-HRP (no. 112-035-175, Jackson Immunoresearch, 1:10,000); mouse α-rat IgG1-HRP (E. Kremmer, Helmholtz Zentrum München, 1:1,000); mouse α-rat IgG2c-HRP (E. Kremmer, Helmholtz Zentrum München, 1:1,000); rat α-mouse IgG2a-HRP (E. Kremmer, Helmholtz Zentrum München, 1:1,000).

### Generation of TMEM129-specific mAbs

TMEM129-specific rat and mouse monoclonal Abs were prepared by immunizing respectively Lou/C rats and CBL mice with OVA-coupled peptides encompassing TMEM129 amino acids 191–204 (VTESRQHELSPDSN). Hybridomas were subsequently generated using standard hybridoma culturing techniques. The following TMEM129-specific mAbs were used: 4G10 of rat subclass IgG1, 13E2 of isotype IgG2c, and 8D7 and 8G9 mAbs both of mouse subclass IgG2a. TMEM129 specificity of these mAbs in immunoblotting and immunoprecipitation experiments is shown in Supplementary Fig. 9a–d.

### Flow cytometry

To assess HLA class I expression levels, cells were first fixed in 0.5% PFA and subsequently washed in FACS buffer (PBS, 0.5% BSA, 0.02% sodium azide). All subsequent staining protocols and washings were performed in FACS buffer. Endogenous HLA-A3 surface expression and eGFP-Myc-HLA-A2 surface expression were assessed by staining with human α-HLA-A3 OK2F3 mAb and mouse α-Myc 9E10-biotin mAb, respectively. Secondary antibodies used were F(ab′)2 fragment goat α-human IgG+IgM(H+L)-PE and streptavidin-BV421, respectively. Afterwards, cells were subjected to flow cytometry acquisition on a FACSCantoII (BD Bioscience). Flow cytometry data were analysed using FlowJo software.

### Immunoprecipitations

Cells were lysed in 1% Triton X-100 lysis buffer (1.0% Triton X-100, 20 mM MES, 100 mM NaCl, 30 mM Tris, pH 7.5) containing 1 mM Pefabloc SC (Roche) and 10 μM Leupeptin (Roche). Cell fragments were pelleted at 12,000*g* for 20 min at 4 °C. Post-nuclear supernatants were incubated for at least 2 h with Protein A or G Sepharose beads (GE Healthcare) and indicated antibodies. After four washes in IP washing buffer (1.0% Triton X-100, 100 mM NaCl; 30 mM Tris, pH 7.5), proteins were eluted in Laemmli sample buffer. Immunoblotting was performed as described below.

### Co-immunoprecipitations

Cells were lysed in 1% Digitonin lysis buffer (1% Digitonin (Calbiochem), 50 mM Tris–HCl, 5 mM MgCl_2_, 150 mM NaCl; pH 7.5) containing 1 mM Pefabloc SC (Roche) and 10 μM Leupeptin (Roche). Lysates were incubated for 90 min at 4 °C. Cell fragments were pelleted 12,000*g* for 20 min at 4 °C. Post-nuclear supernatants were incubated overnight with StrepTactin beads (GE Healthcare), or FLAG-M2-coupled beads (Sigma). After four washes in 1% digitonin lysis buffer, proteins were eluted in elution buffer (For StrepTactin beads: 2.5 mM desthiobiotin, 150 mM NaCl, 100 mM Tris–HCl, 1 mM EDTA, pH 8; For FLAG-M2-coupled beads: 500 μg ml^−1^ FLAG peptide, 150 mM NaCl, 100 mM Tris–HCl, pH 7.5) for 30 min on ice. Beads were pelleted, the supernatant was transferred to a new tube and subsequently denatured in Laemmli sample buffer containing DTT. Immunoblotting was performed as described below.

### Immunoblotting

Samples were incubated at 95 °C for 5 min, separated by SDS–PAGE and proteins were transferred to PVDF membranes (Immobilon-P, Millipore). Membranes were probed with indicated antibodies. Reactive bands were detected by ECL (Thermo Scientific Pierce), and exposed to Amersham Hyperfilm ECL films (GE Healthcare). Full scans of all western blots are provided in Supplementary Fig. 11.

### *In vitro* ubiquitination assay

Cells were lysed in 1% Triton X-100 lysis buffer (1.0% Triton X-100, 20 mM MES, 100 mM NaCl, 30 mM Tris, pH 7.5) containing 1 mM Pefabloc SC (Roche) and 10 μM Leupeptin (Roche). Cell fragments were pelleted at 12,000*g* for 20 min at 4 °C. Post-nuclear supernatants were incubated overnight with StrepTactin beads (GE Healthcare). After four washes in stringent IP washing buffer (1.0% Triton X-100, 400 mM NaCl, 20 μM ZnSO_4_, 30 mM Tris, pH 7.5), proteins were eluted in elution buffer (2.5 mM desthiobiotin, 150 mM NaCl, 20 μM ZnSO_4_, 100 mM Tris–HCl, pH 8) for 30 min on ice. Beads were pelleted, and the supernatant was directly used in the *in vitro* ubiquitination assay. The MuRF1/S5a ubiquitination kit (no. K-102) was purchased from Boston Biochem, and reactions were performed as described by the manufacturer. To test the E3 ubiquitin ligase activity of TMEM129, GST-MuRF1 was replaced by immunoprecipitated TMEM129(ΔRING)-FLAG-ST2. Immunoblotting was performed as described above.

### Pulse-chase analysis

To study HLA class I dislocation, cells were pre-incubated with 20 μM MG132 (Sigma-Aldrich) for 4 h. All subsequent steps were performed in the presence of 20 μM MG132. To study HLA class I degradation, cells were not treated with a proteasome inhibitor. Cells were starved for 30 min in Met- and Cys-free medium, and pulsed for 10 min with EasyTag EXPRESS [^35^S] Protein Labeling Mix (20 Mbq per 10 × 10^6^ cells ml^−1^; Perkin-Elmer). Cells were chased for the indicated time periods and lysed in 1% Triton X-100 lysis buffer. HLA class I HCs were immunoprecipitated as described above. Samples were incubated at 95 °C for 5 min, separated by SDS–PAGE (12%) and bands were visualized by exposure to a BioMAX MR High Resolution film (Kodak).

### Fluorescence confocal microscopy

MelJuSo cells were grown overnight on 12 mm circular glass coverslips. Attached cells were washed with PBS supplemented with 0.5 mM MgCl_2_ and 1 mM CaCl_2_ (PBS++) and fixed in 3% paraformaldehyde for 15 min at room temperature. Coverslips were washed again in PBS++ and embedded on microscope slides with Mowiol 4-88 (Carl Roth, Germany). Slides were air-dried overnight at room temperature and imaged using a Leica TCS SP5 confocal microscope equipped with a HCX PL APO CS × 63/1.40–0.60 OIL objective (Leica Microsystems, the Netherlands). eGFP and mCherry fluorescent signals were detected with PMTs set at the appropriate bandwidth after excitation using the 488 nm argon laser for eGFP and the 543 nm helium neon laser for mCherry. Images were processed and analysed using Leica SP5 software.

### UPR assessment

Cells were either incubated with Thapsigargin (50 μM) or DMSO. After 6 h of incubation, cells were harvested and mRNA was isolated using standard Trizol RNA isolation procedures. Possible genomic DNA contamination was removed using a TURBO DNase kit (Life Technologies) according to the manufacturer’s protocol. Using oligo dT primers, mRNA was specifically reverse transcribed into cDNA (SuperScriptIII, Invitrogen) according to the manufacturer’s protocol. GAPDH and XBP-1 were then amplified using specific primers (Supplementary Table 3) and DreamTaq Green (Fermentas). PCR products were separated in SYBRsafe (Life Technologies)-containing 2.5% agarose gels.

## Author contributions

The work presented here was carried out in collaboration between all authors. M.L.v.d.W., R.J.L. and E.J.H.J.W. designed the project, organized the research and wrote the manuscript. M.L.v.d.W. performed all experiments, except for the genome-wide shRNA library screen and the COPI validation experiments. R.J.L. designed the CRISPR/Cas-mediated genome engineering experiments, and R.J.L. and M.L.v.d.W. performed these experiments. R.J.L. designed and performed DNA cloning experiments. R.J.L. and C.M.V. performed the genome-wide shRNA library screen. R.J.L., M.C.B., J.S.W. and M.T.M. designed the genome-wide shRNA library and R.J.L., M.C.B. and W.P. cloned and performed QC of the shRNA library. E.M.L. and S.C. provided essential reagents to construct the shRNA library. M.C.B. analysed the data from the shRNA library screen. M.A.M.L. performed the COPI validation experiments and R.D.L. performed the fluorescence microscopy experiments. H.H. provided technical assistance with the pulse-chase experiments. E.K. generated the TMEM129 monoclonal antibodies. R.C.H. provided essential shRNA expression vectors. M.E.R. gave conceptual advice.

## Additional information

**How to cite this article:** van de Weijer, M. L. *et al.* A high-coverage shRNA screen identifies TMEM129 as an E3 ligase involved in ER-associated protein degradation. *Nat. Commun.* 5:3832 doi: 10.1038/ncomms4832 (2014).

## Supplementary Material

Supplementary Information Supplementary Figures 1-11, Supplementary Tables 1-3 and Supplementary References

## Figures and Tables

**Table 1:** Overlapping genes in top 100 from duplicate screens.

**Gene ID**	**Gene symbol**	**Full name**
372	ARCN1	Archain 1
1314	COPA	Coatomer protein complex, subunit alpha
1315	COPB1	Coatomer protein complex, subunit beta 1
1939	EIF2D	Eukaryotic translation initiation factor 2d
5684	PSMA3	Proteasome (prosome, macropain) subunit, alpha type, 3
5687	PSMA6	Proteasome (prosome, macropain) subunit, alpha type, 6
5688	PSMA7	Proteasome (prosome, macropain) subunit, alpha type, 7
5695	PSMB7	Proteasome (prosome, macropain) subunit, beta type, 7
5701	PSMC2	Proteasome (prosome, macropain) 26s subunit, atpase, 2
5702	PSMC3	Proteasome (prosome, macropain) 26s subunit, atpase, 3
5704	PSMC4	Proteasome (prosome, macropain) 26s subunit, atpase, 4
5705	PSMC5	Proteasome (prosome, macropain) 26s subunit, atpase, 5
5707	PSMD1	Proteasome (prosome, macropain) 26s subunit, non-atpase, 1
5708	PSMD2	Proteasome (prosome, macropain) 26s subunit, non-atpase, 2
5709	PSMD3	Proteasome (prosome, macropain) 26s subunit, non-atpase, 3
6633	SNRPD2	Small nuclear ribonucleoprotein d2 polypeptide 16.5 kda
7415	VCP	Valosin-containing protein
9276	COPB2	Coatomer protein complex, subunit beta 2 (beta prime)
22818	COPZ1	Coatomer protein complex, subunit zeta 1
22820	COPG	Coatomer protein complex, subunit gamma
92305	TMEM129	Transmembrane protein 129

Entrez gene IDs, gene symbols and full names of genes present in the top 100 of two independent screens are presented. All genes validated in subsequent shRNA validation experiments, except for EIF2D and SNRPD2. Genes are ranked according gene ID number.
