# Supplementary material for: A high-coverage shRNA screen identifies TMEM129 as an E3 ligase involved in ER-associated protein degradation
Source: Nat Commun. 2014 May 8;5:3832. doi: 10.1038/ncomms4832 (PMC4024746; doi:10.1038/ncomms4832)
Supplement: Supplementary Information — Supplementary Figures 1-11, Supplementary Tables 1-3 and Supplementary References [file ncomms4832-s1.pdf]

SUPPLEMENTARY INFORMATION

A high-coverage shRNA screen identifies TMEM129 as an E3 ligase involved in ER-associated protein degradation

Michael L. van de Weijer[1], Michael C. Bassik[2,7], Rutger D. Luteijn[1], Cornelia M. Voorburg[1], Mirjam A.M. Lohuis[1], Elisabeth Kremmer[3], Rob C. Hoeben[4], Emily M. LeProust[5,7], Siyuan Chen[5,7], Hanneke Hoelen[1], Maaike E. Ressing[1,4], Weronika Patena[2,6,7], Jonathan S. Weissman[2], Michael T. McManus[6,*], Emmanuel J.H.J. Wiertz[1,8,*], and Robert Jan Lebbink[1,8,*].

[1]Medical Microbiology, University Medical Center Utrecht, Utrecht 3584CX, The Netherlands; [2]Department of Cellular and Molecular Pharmacology, California Institute for Quantitative Biomedical Research, and Howard Hughes Medical Institute, University of California, San Francisco, California 94158, USA; [3]Helmholtz Zentrum München, German Research Center for Environmental Health, Institute of Molecular Immunology, 81377 Munich, Germany; [4]Department of Molecular Cell Biology, Leiden University Medical Center, Leiden 2333ZC, The Netherlands; [5]Genomics Solution Unit, Agilent Technologies Inc., Santa Clara, California 95051, USA; [6]Department of Microbiology and Immunology, University of California, San Francisco, California 94143, USA. [7]Present address: M.C.B., Department of Genetics, Stanford University, California 94305, USA; E.M.L. and S.C., Twist Bioscience, San Francisco, CA 94158, USA; W.P., Carnegie Institution for Science, Department of Plant Biology, Stanford, California 94305, USA. [8]These authors contributed equally to this work.

[*]Correspondence: M.T.M. (michael.mcmanus@ucsf.edu), E.J.H.J.W. (E.Wiertz@umcutrecht.nl), or R.J.L. (R.J.Lebbink-2@umcutrecht.nl).

Overview of Supplementary Information

Supplementary Information provides 11 Supplementary Figures and 3 Supplementary Tables; a brief description for each element is given below.

- Supplementary Figure 1 shows the extended results for all four TMEM129-targeting shRNAs used throughout this study.
- Supplementary Figure 2 provides a detailed flow cytometry analysis of TMEM129-null cell clones generated via CRISPR/Cas genome engineering.
- Supplementary Figure 3 shows a pulse-chase analysis of HLA class I in the absence or presence of HCMV US11.
- Supplementary Figure 4 provides analysis of the COPI hits.
- Supplementary Figure 5 shows the evolutionary conservation of TMEM129.
- Supplementary Figure 6 shows the state of the UPR in TMEM129-null cells.
- Supplementary Figure 7 provides a functional assessment of HRD1 in US11-mediated HLA class I downregulation.
- Supplementary Figure 8 provides an overview of genomic target sites for the gRNAs used throughout this study.
- Supplementary Figure 9 shows the validation of generated TMEM129-specific mAbs.
- Supplementary Figure 10 shows the functionality of the TMEM129 and US11 constructs used throughout this study.
- Supplementary Figure 11 shows the full scans of the Western blots.
- Supplementary Table 1 shows sequences of shRNAs used for independent validation experiments.
- Supplementary Table 2 shows gRNA sequences used for CRISPR/Cas-mediated gene disruption.
- Supplementary Table 3 shows primer sequences used for UPR assessment.

Supplementary Figure 1

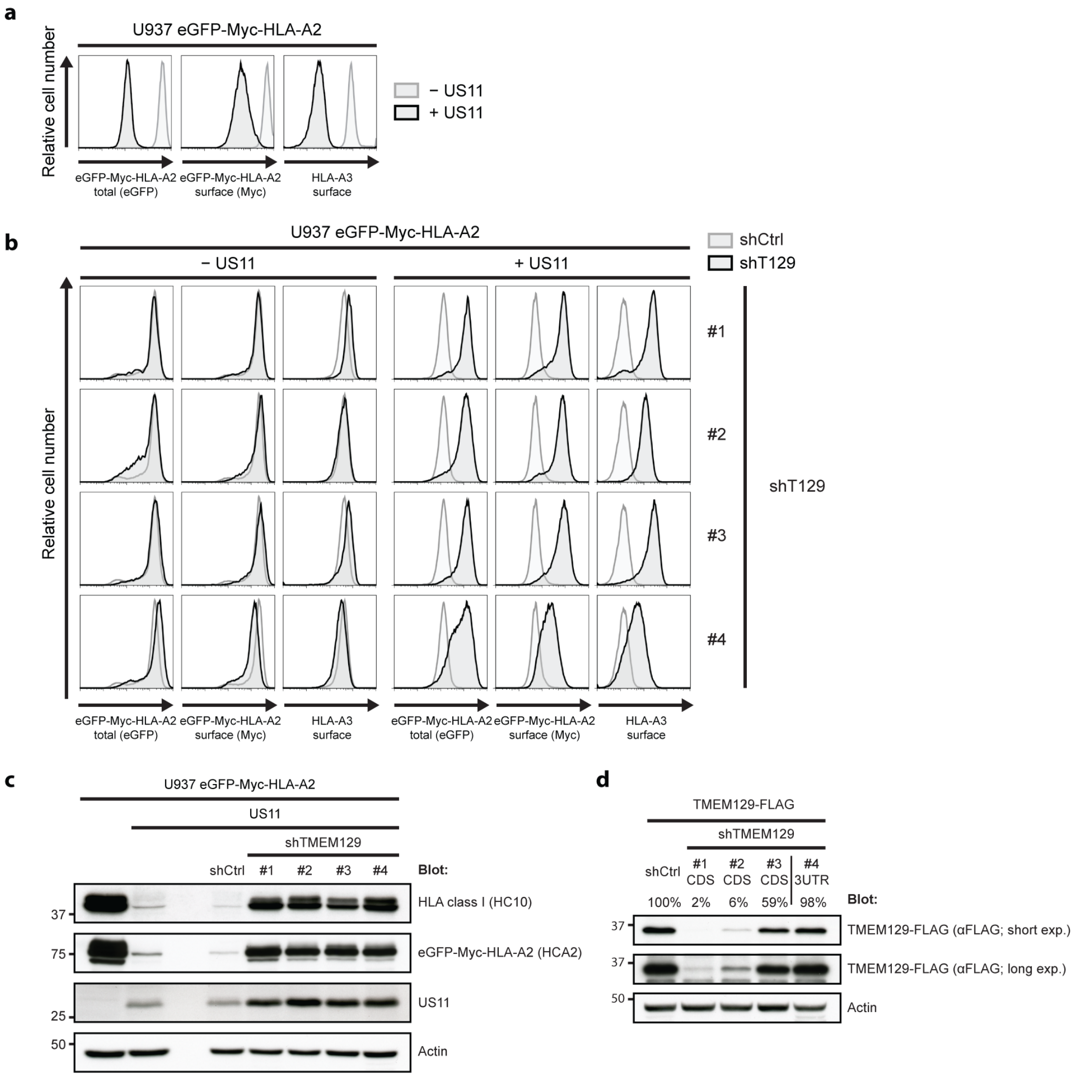

Supplementary Figure 1: TMEM129 is crucial for US11-mediated HLA class I downregulation.

(a) HCMV US11 induces downregulation of the eGFP-Myc-HLA-A2 chimaera and endogenous HLA class I in U937 cells. Flow cytometry analysis of total (eGFP) and surface (Myc) expression of eGFP-Myc HLA-A2 and surface expression of endogenous HLA-A3 in U937 eGFP-Myc-HLA-A2 cells with (black histogram) and without expression of HCMV US11 (gray histogram). **(b)** Depletion of endogenous TMEM129 by shRNAs induces potent rescue of eGFP-Myc-HLA-A2 and endogenous HLA-A3 expression in U937 eGFP-Myc-HLA-A2 US11 cells. Four shRNAs targeting TMEM129 (black histograms) or one control shRNA (gray

histogram) were introduced in U937 eGFP-Myc-HLA-A2 cells and U937 eGFP-Myc-HLA-A2 US11 cells. 7 dpi flow cytometry analysis was performed of endogenous surface HLA-A3, and surface (Myc) and total (eGFP) eGFP-Myc-HLA-A2. **(c)** Immunoblot analysis of endogenous HLA class I, eGFP-Myc-HLA-A2, US11, and loading control actin in U937 eGFP-Myc-HLA-A2 US11 cells after mock (shCtrl) or TMEM129 (shTMEM129 #1-4) depletion. **(d)** Downregulation of ectopically expressed tagged TMEM129 using shRNAs. Immunoblot analysis of TMEM129-FLAG levels and loading control actin in U937 cells after mock (shCtrl) or TMEM129 (shT129 #1-4) depletion 7 dpi. Percentages indicate expression levels compared to mock (shCtrl) depletion normalized to actin levels. Of note, effective shRNA-mediated depletion was confirmed in cells ectopically expressing TMEM129-FLAG, as endogenous protein levels of TMEM129 could not be assessed via immunoblotting. Therefore, in this experimental setup, the fourth shRNA had no effect on TMEM129-FLAG expression levels (lane 5), because this shRNA targets the 3'-UTR, which is absent in the ectopic TMEM129-FLAG construct. In all further experiments, where TMEM129 is depleted, the most efficient and stable TMEM129-targeting shRNA (shTMEM129 #1) was used. CDS, coding sequence; 3UTR, 3' untranslated region.

Supplementary Figure 2

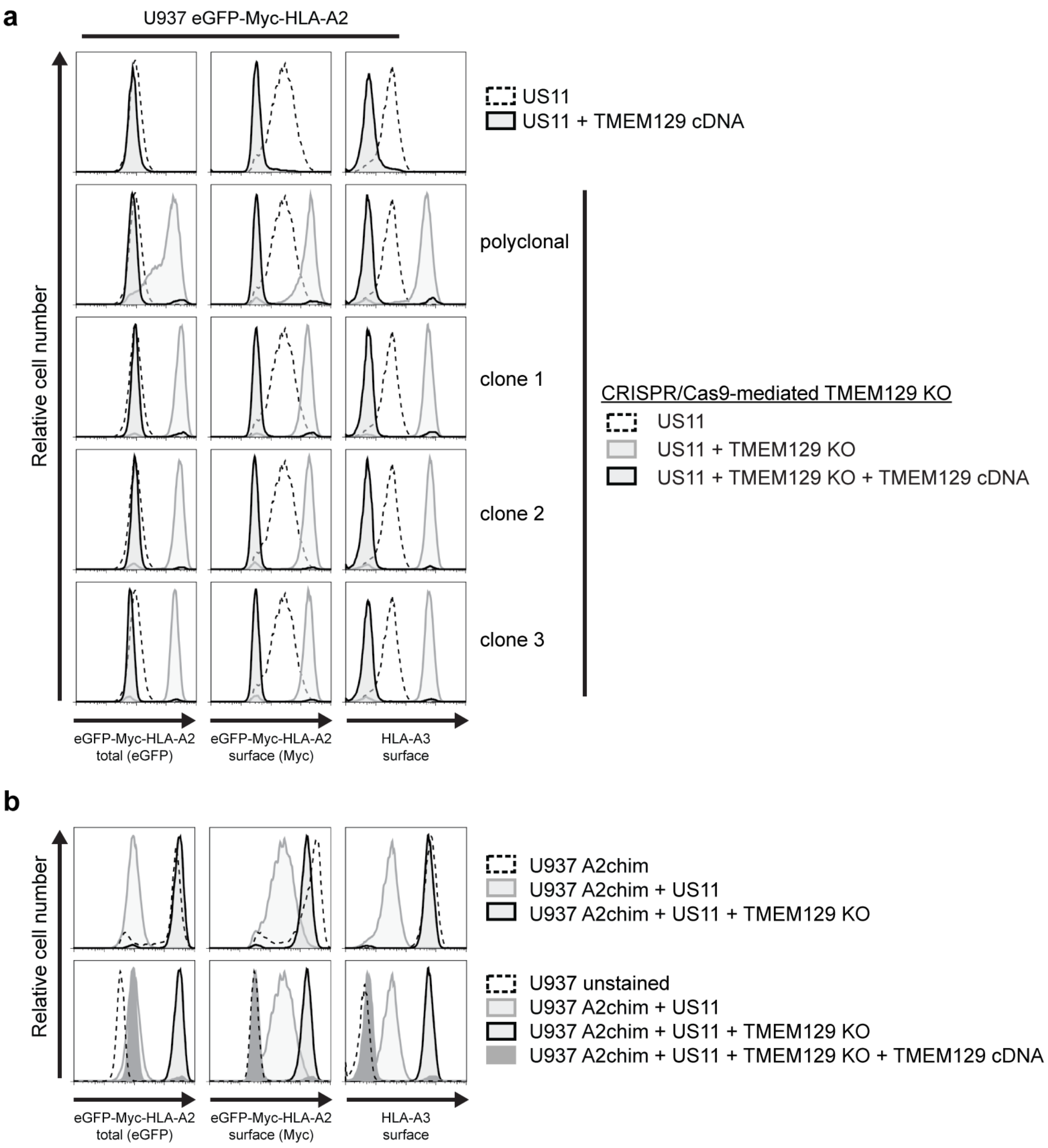

Supplementary Figure 2: Generation and analysis of CRISPR/Cas-mediated TMEM129-null lines.

(a) CRISPR/Cas-mediated knockout of TMEM129 induces potent rescue of HLA class I. U937 eGFP-Myc-HLA-A2 US11 cells were transiently transfected with Cas9- and gTMEM129-expressing plasmids. Cells with increased eGFP signal were sorted (polyclonal), and subsequently clonally expanded, which resulted in the generation of 3 individual clones (clone 1-3). TMEM129 was re-introduced in these cells. Cells were then subjected to flow cytometry analysis of surface endogenous HLA-A3, and surface (Myc)

and total (eGFP) eGFP-Myc-HLA-A2. **(b, upper panel)** TMEM129-null US11 cells show completely restored HLA class I expression. Surface endogenous HLA-A3, and surface (Myc) and total (eGFP) eGFP-Myc-HLA-A2 comparison between U937 eGFP-Myc-HLA-A2 (dashed histograms), U937 eGFP-Myc-HLA-A2 US11 (gray-lined histograms), and U937 eGFP-Myc-HLA-A2 US11 TMEM129-null cells (black-lined histograms). **(b, lower panel)** TMEM129-overexpressing TMEM129-null US11 cells are almost devoid of HLA class I molecules. Surface endogenous HLA-A3, and surface (Myc) and total (eGFP) eGFP-Myc-HLA-A2 comparison between unstained U937 (dashed histograms), U937 eGFP-Myc-HLA-A2 US11 (gray-lined histograms), and U937 eGFP-Myc-HLA-A2 US11 TMEM129-null cells (black-lined histograms), and U937 eGFP-Myc-HLA-A2 US11 TMEM129-null cells overexpressing TMEM129 cDNA (gray-filled histograms).

Supplementary Figure 3

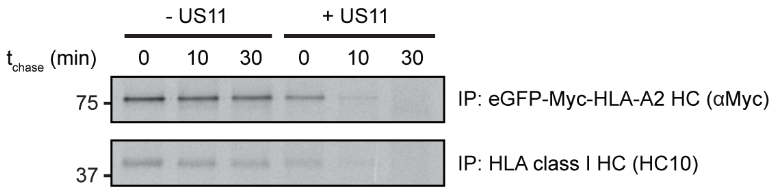

Supplementary Figure 3: US11 induces rapid degradation of HLA class I in U937 cells.

U937 eGFP-Myc-HLA-A2 cells with and without US11 were subjected to pulse chase analysis, for which cells were radioactively labeled for 10 min, and chased for the indicated timeframes. Subsequently, eGFP-Myc-HLA-A2 and HLA class I HCs were immunoprecipitated from lysates using respectively the Myc-specific 9E10 mAb and the HLA class I HC-specific HC10 mAb.

Supplementary Figure 4

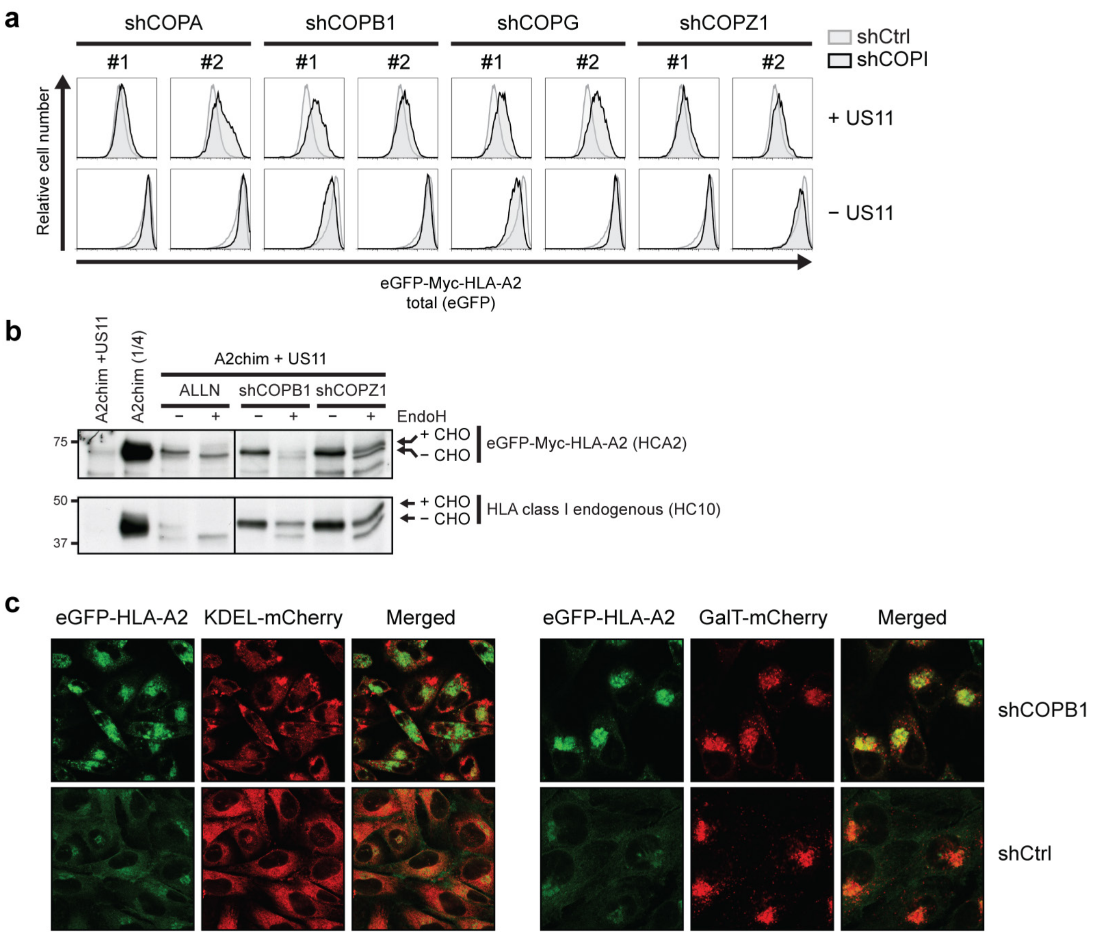

Supplementary Figure 4: COPI depletion causes accumulation of HLA class I in the Golgi.

(a) COPI subunit depletion by shRNAs induces rescue of chimaeric HLA-A2 expression in U937 cells co-expressing eGFP-Myc-HLA-A2 and US11. shRNAs targeting indicated COPI subunits (black histograms) or control shRNAs (gray histogram) were introduced in U937 eGFP-Myc-HLA-A2 and U937 eGFP-Myc-HLA-A2 US11 cells, and total expression of eGFP-Myc-HLA-A2 was assessed by measuring eGFP expression 4 dpi. by flow cytometry, whereas surface expression of eGFP-Myc-HLA-A2 and endogenous HLA-A3 were assessed by specific antibody stainings (anti-Myc and anti-A3 respectively). Of note, as COPI subunit depletion induced rapid cell death (approximately 4 dpi), the full effect of COPI depletion could not be assessed since optimal protein target knockdown is often only reached after approximately 6 dpi.[1]. **(b)**

COPI subunit depletion by shRNAs induces rescue of HLA class I to post-ER compartments in U937 cells co-expressing eGFP-Myc-HLA-A2 and US11. Lysates of U937 eGFP-Myc-HLA-A2 US11 cells incubated with either DMSO or ALLN, or U937 eGFP-Myc-HLA-A2 US11 cells expressing an shRNA targeting either COPB1 or COPZ1, were mock- or EndoH-treated, after which immunoblot analysis was performed to detect endogenous HLA class I and eGFP-Myc-HLA-A2. Onefourth of U937 eGFP-Myc-HLA-A2 lysate as compared to other lanes is loaded as a reference. **(c)** COPI subunit depletion causes accumulation of eGFP-Myc-HLA-A2 in the Golgi-compartment. MJS GFP-HLA-A2 cells stably expressing either mCherry-KDEL (ER-localized) or GAIT-mCherry (Golgi-localized) were either mock- or COPI-subunit-depleted and fixed, after which immunofluorescence analysis for eGFP-HLA-A2 and mCherry-KDEL or GAIT-mCherry was performed. Individual images were merged to assess co-localization.

Supplementary Figure 5

```
H.sapiens          -------------------MDSPEVTFTLAYL----------VFAVCFVFTPNEF-HAAG     30
M.musculus         -------------------MDSPEVTFTLAYL----------VFAVCFVFTPNEF-YSAG     30
D.rerio            -------------------MDRPDATFTLAYV----------VFALCFVFTPNEF-RSAG     30
D.melanogaster     -------------------MDESELLFNLFYI----------LLCMVIIYPPEEF-QRLG     30
C.elegans          MTSIKRGTWFFIFVLGVFTAITCEFTTQLTYLHVVSHIAILTAAHFALVFPPEELLDGLG     60
                                          :    * *:             . ::: *:*:    *

H.sapiens          LTVQNLLSGWLGSEDAAFVPFHLRRTAATLLCHSLLP---LGYYVGMCLAASE----KRL     83
M.musculus         LTVQNLLSGWLGSEDAAFVPYHLRRTSATLLCHSLLP---LGYYMGMCFAASE----KQL     83
D.rerio            FTVQHMFSEWLGSEDISFIQHHIRRTTLTVLFHSFLP---LGYYIGMCFAAPE----QNL     83
D.melanogaster     FTIEQLFARFLGEEYLDFVGYHQRRTSLNLFVHSCLP---FSYFLIHRLKFSVFATQEPL     87
C.elegans          LTLFDLFTILYNSRQHDFVEYAAKRVAFFHAFVAFYPCVIAAYFVHG----------NIL     110
                   :*: .:::    ..   *: .  :*.:      :  *    .*::            : *

H.sapiens          H-------------ALSQAPEAWRLFLLLAVTLPSIACILIYYWSRDRWACHPLARTLAL     130
M.musculus         Y-------------SPGQAPEAWQLFLLLAVTLPLLSCTLIYYWSWDRWTRHPLAQTLAL     130
D.rerio            M-------------YVHHASQGWQMYFGLSLVIQLLSCALAFYWSRRGWANHPICKALSV     130
D.melanogaster     EDFDLDPDFPMPQEAVAFKTLTWKTAQRFSVLAVLAMPALIFNWHQENWRRHPISKALSK     147
C.elegans          MTAADTRRFP----SVLDYPWMWFTVLFVALIANGYYCIRI-----KDYKKWPIYQY---     158
                                         *     .::                 :   *: :

H.sapiens          YALPQSGWQAVASSVNTEFRRIDKFATGAP-GARVIVTDTWVMKVTTYRVHVAQQQDVHL     189
M.musculus         YALPQSGWQAVASSINTEFRRIDKFATGAP-GARVIVTDTWVMKVTTYRVHVAQQQDVHL     189
D.rerio            HALPQSSWRAVASSINTEFRRIDKFASGSP-SARVIVTDTWVMKVTTYSLHVALHQDCHL     189
D.melanogaster     YSITPGSYSAVASEIGIEFRQPEIYKKKLNSISSVIATQNWIIKTTMYNVHFAHQSNTSL     207
C.elegans          -YIDNP---DCVNEAQTVIADPETFRIKLSQRSFLFVTRNFLVYTSNWRFMAVKLADVRL     214
                     :        ...    :   : :       : ::.* .::: .: : .  .   :  *

H.sapiens          TVTESRQHELS-PDSNLPVQLLTIRVASTNPAVQAFDIWLNSTEYGELCEKLRAPIRRAA     248
M.musculus         TVTESRQHDLS-PDSNLPVQLLTIRVASTSPGTQPFDIRLNSSEYGELCEKLHAPIRSAA     248
D.rerio            TVTDSKHHSLS-PDLNTPVQIVTITVGSINPRVKSFDIRLKSTEYAELQEKLHAPIRNAA     248
D.melanogaster     SVAKAETYNISHQDQNDTLQMISIIVRPMRQGVSDFHIRINALEFRNLENRVRRPIAIPS     267
C.elegans          QVDNTRMPMLPNQDEEERIRSIFVKVSFRPAYITPFTITLRQDYYRQLNEILEVPIFVPP     274
                    * .:.   :   * :  :: : : *         * * :.   : :* : :. **

H.sapiens          HVVIHQSLGDLFLETFASLVEVNPAY-SVPSSQELEACIGCMQTRASVKLVKTCQEAA--     305
M.musculus         NVVIRQSLGDLFLETFASHVEVNPAY-SVPSNQELEPCIGCMQTRASVKLVKTCQEPA--     305
D.rerio            NVVIHLTMSELFLETFKSYVRMNVVY-KCPSGQELEPCIGCMQVNANVKLLCLCQS-D--     304
D.melanogaster     NIQLHRNVIDRFVDVFKAQVAQNPIF---PDDATTEKCFACMLNEPNTKIHKQCADFDRN     324
C.elegans          HINVPLTFMEELKEDFIQRIASNTRVTHRVKASEKDPCFACGTEENMVKIERSCDGQEQR     334
                   .: :  .. : : : *   :  *       .    : *:.*   .  .*:   *

H.sapiens          ----------TGECQQCYCRPMWCLTCMGKWFASRQDPL--RPDTWLASRVPCPTCRARF     353
M.musculus         ----------VGECQQCYCRPMWCLTCMGKWFASRQDPQ--RPDTWLASRVPCPTCRARF     353
D.rerio            ----------EGECQQCYCRPMWCLTCMGKWFASRQDQQ--QPETWLSSRVPCPTCRAKF     352
D.melanogaster     G----APLANGACCSNCYCRPMWCVECLARWFAARQSDV--DREVWLEQKCTCPMCRAKF     378
C.elegans          VFFHDIGARFTPPCENCICRPLWCRSCLAQIFIGRQNIDNVYRYEYHRGSAQCPTCRKNF     394
                                *.:* ***:**  *:.: * .**.        :      ** ** .*

H.sapiens          CILDVCTVR----------------------     362
M.musculus         CILDVCCVR----------------------     362
D.rerio            CILDVCPIE----------------------     361
D.melanogaster     CVLDVSYITTTTVDATNSTQSQGTEEDTNDTT     409
C.elegans          CIRDVHCVDFDYISEEN--------------     411
                   *: **  :
```

% identity					
Hs	100				
Mm	89.2	100			
Dr	64.8	66.5	100		
Dm	32.8	33.6	34.7	100	
Ce	24.7	25	22.7	23.9	100
	Hs	***Mm***	***Dr***	***Dm***	***Ce***

Supplementary Figure 5: Multiple sequence alignment of TMEM129. Amino acid sequences of TMEM129 from *H. sapiens* (UniProt: A0AVI4), *M. musculus* (UniProt: Q8K304), *D. rerio* (UniProt: Q6PD82), *D. melanogaster* (UniProt: Q9VN16), and *C. elegans* (UniProt: O17638) were aligned using ClustalO software, and conserved residues were visualized using JalView. Identity percentages were calculated using Clustal software. Green: fully conserved residue; Yellow, 50% or more residues with similar identity; Red: fully conserved zinc-coordinating cysteine as part of the RING domain. An '*' (asterisk) indicates a single, fully conserved residue. A ':' (colon) indicates conservation between groups of strongly similar properties with a scoring of > 0.5 in the Gonnet PAM 250 matrix. A '.' (period) indicates conservation between groups of weakly similar properties with a scoring of =< 0.5 in the Gonnet PAM 250 matrix.

Supplementary Figure 6

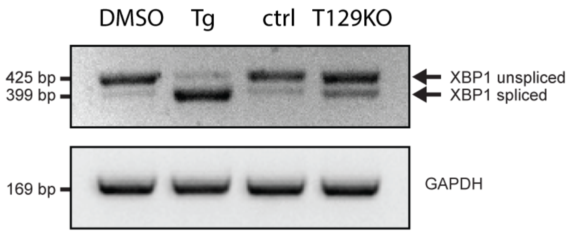

Supplementary Figure 6: TMEM129 knockout does not induce a strong UPR response. RNA was isolated from TMEM129-null cells (T129KO), generated via the CRISPR/Cas9 system, or from control cells (ctrl). From the RNA, mRNA was specifically converted to cDNA, after which levels of spliced and unspliced XBP-1 were detected via semi-quantitative PCR as a marker for UPR activation. GAPDH was included as a loading control. As a positive UPR control, cells were incubated with Thapsigargin (50 µM) for 6 hours, or, as a negative control, with DMSO.

Supplementary Figure 7

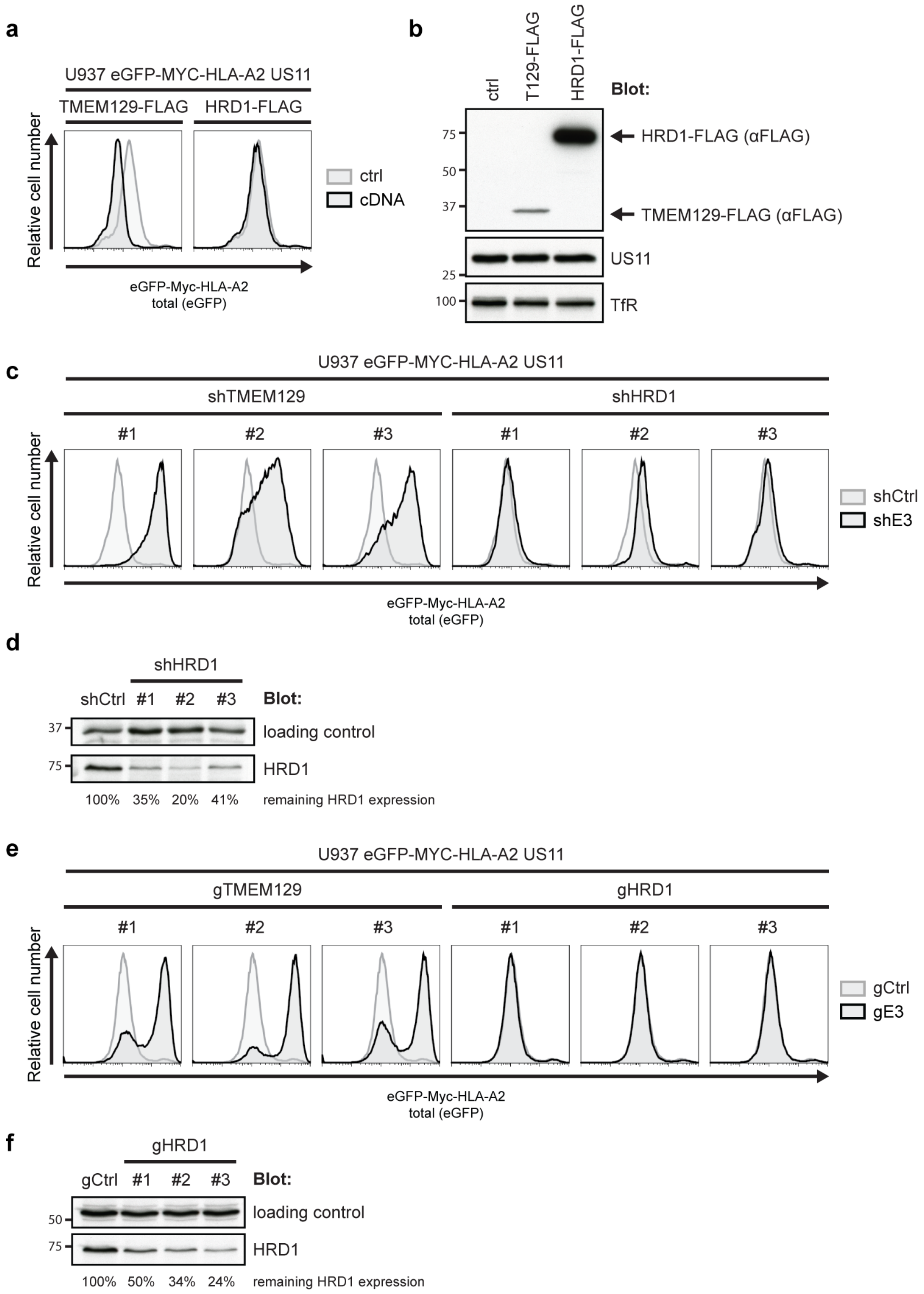

Supplementary Figure 7: Functional assessment of HRD1 in US11-mediated HLA class I downregulation.

(a) HRD1 overexpression does not enhance US11-mediated HLA class I downregulation**.** Flow cytometry analysis of total (eGFP) eGFP-Myc-HLA-A2 in U937 eGFP-Myc-HLA-A2 US11 cells overexpressing TMEM129-FLAG (black-lined histogram, left panels), HRD1-FLAG (black-lined histogram, right panels), or a control vector (gray-lined histograms, both panels). **(b)** Immunoblot analysis of TMEM129-FLAG and HRD1-FLAG expression levels in cells used in (a). US11 expression levels are indicated as well, along with transferrin receptor used as a loading control. **(c)** ShRNA-mediated HRD1 depletion does not rescue HLA class I from US11-mediated downregulation. Flow cytometry analysis of total (eGFP) eGFP-Myc-HLA-A2 in U937 eGFP-Myc-HLA-A2 US11 cells expressing shRNAs targeting TMEM129 (black-lined histograms, left three panels) or HRD1 (black-lined histograms, right three panels), or a control shRNA (gray-lined histograms). **(d)** Assessment of knockdown efficiency of HRD1-targeting shRNAs. A background band was used as a loading control. Percentages indicate HRD1 expression levels as compared to mock (shCtrl) depletion normalized against loading control levels. **(e)** CRISPR/Cas-mediated *HRD1* gene disruption does not rescue HLA class I from US11-mediated downregulation. Same experimental setup as in C, but instead of shRNAs, CRISPR/Cas gRNAs were lentivirally introduced. **(f)** Validation of *HRD1*-targeting gRNAs. Western blots depict the expression level of HRD1 in the indicated polyclonal CRISPR/Cas-mediated HRD1 knock-out or control cells. The lentiviral delivery of the CRISPR/Cas system results in a mixed population of cells in which either no, one or both *HRD1* alleles are knocked-out. Typically, we observe approximately 30-90% of full knock-out phenotypes upon CRISPR/Cas mediated genome editing. Indicated percentages show HRD1 expression levels in these polyclonal lines as compared to control gRNA treated cells normalized to a loading control.

Supplementary Figure 8

TMEM129 (NM_001127266)

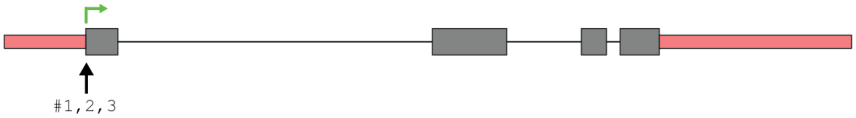


```
GS:   AGGTGACCTTCACTCTCGCCTATCTGGTGTTCGCCGTGTGCTTCGTGTTCACGCCCAACGAGTTCCACGCGGCGGGGC
g#1:      GACCTTCACTCTCGCCTATCTGG
g#2*:                   CCTATCTGGTGTTCGCCGTGTGC
g#3:                                                      GCCCAACGAGTTCCACGCGGCGG
```

HRD1 (NM_172230)

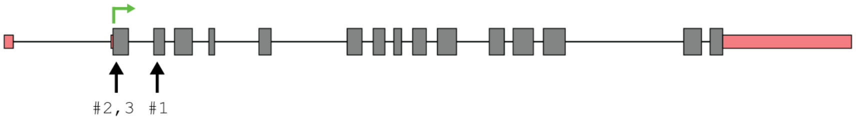


```
GS    CAAGGTGATGGGCAAGGTGTTCTTTGGGCAA
g#1:      GTGATGGGCAAGGTGTTCTTTGG

GS    GATGGCGGCCAGCCTGGCGCTGACCGGGGCT
g#2:      GCGGCCAGCCTGGCGCTGACCGG

GS    TCCGGGCCAGGGCAATGTTCCGCACGGCAGT
g#3:      GGCCAGGGCAATGTTCCGCACGG
```

UBE2J1 (NM_016021)

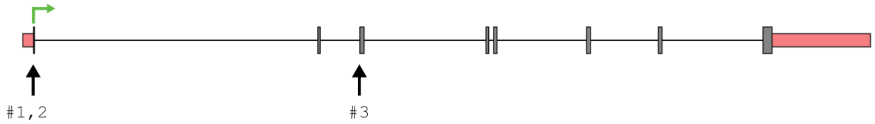


```
GS    CAAGCCAGCGACCCACCATGGAGACCCGCTACAACCTGA
g#1*:     CCAGCGACCCACCATGGAGACCC
g#2*:             CCACCATGGAGACCCGCTACAAC

GS    TTTTGAATGGCACTTCACGGTTAGAGGGCCC
g#3:      GAATGGCACTTCACGGTTAGAGG
```

UBE2J2 (NM_194315)

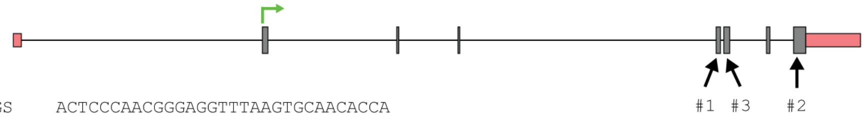


```
GS    ACTCCCAACGGGAGGTTTAAGTGCAACACCA
g#1*:     CCAACGGGAGGTTTAAGTGCAAC

GS    CGCACCTCGTCCAGAACGGGATTCAGCTGCT
g#2*:     CCTCGTCCAGAACGGGATTCAGC

GS    GCAGTATAGAGACGTCGGACTTCACGGTAAG
g#3:      TATAGAGACGTCGGACTTCACGG
```

UBE2K (NM_005339)

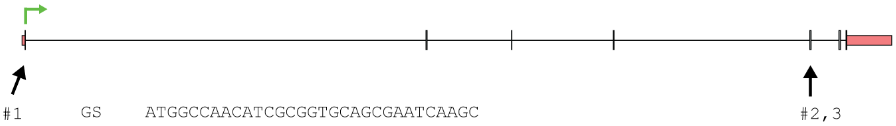


```
GS    ATGGCCAACATCGCGGTGCAGCGAATCAAGC
g#1*:     CCAACATCGCGGTGCAGCGAATC

GS    CTCTCCGCACGGTATTATTGTCATTGCAAGC
g#2*:     CCGCACGGTATTATTGTCATTGC

GS    CACAGGATGCTGTAGTAGCAAATCAGGTAAG
g#3:      GGATGCTGTAGTAGCAAATCAGG
```

Legend

Intron: —

UTR:

Exon:

Start codon:

Supplementary Figure 8: Genomic target sites of gRNAs for CRISPR/Cas-mediated genome engineering.

gRNAs were designed to target coding regions of *TMEM129*, *HRD1*, *UBE2J1*, *UBE2J2*, and *UBE2K* that lie as close to the start codon as possible to minimize potential expression of truncated gene products. However, gRNA target sites were limited by the presence of the PAM motive (underlined) and were designed to be uniquely present in the human genome to limit the impact of potential off-targeting effects[2]. gRNA target sites that are indicated with an asterisk, are reverse complement to gRNA sequences used. Gene structures including UTRs, introns and exons have been visualized using FancyGene[3]. See Supplementary Table 2 for more information on the gRNAs that were used in this study. GS, Genomic Sequence; PAM, protospacer adjacent motive; g#, gRNA number.

Supplementary Figure 9

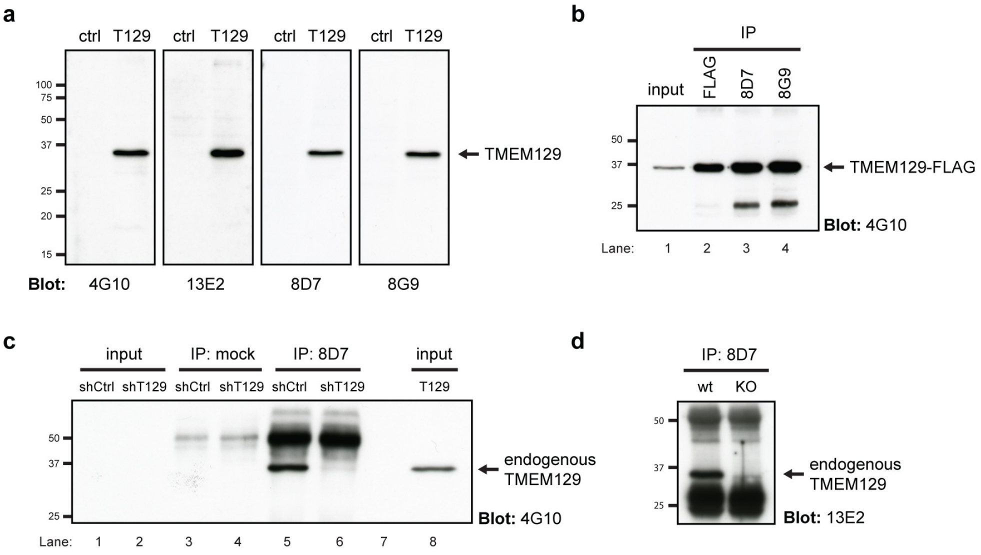

Supplementary Figure 9: TMEM129-specific monoclonal antibodies.

(a) The rat 4G10 and 13E2 mAbs, and the mouse 8D7 and 8G9 mAbs were tested for their TMEM129 specificity using lysates of wildtype U937 cells and TMEM129-overexpressing U937 cells. **(b)** The mouse 8D7 and 8G9 mAbs were tested for their ability to immunoprecipitate TMEM129 from lysates of U937 TMEM129-FLAG cells (lane 3, 4). As a positive immunoprecipitation control, an anti-FLAG immunoprecipitation was included (lane 2). Immunoprecipitated samples were subjected to immunoblotting using the TMEM129-specific 4G10 mAb to visualize TMEM129-FLAG. **(c)** Endogenous TMEM129 can be visualized by immunoprecipitation and subsequent immunoblotting using a TMEM129-specific mAb. Lysates of U937 cells that were either mock- (shCtrl) or TMEM129-depleted (shT129), were subjected to immunoprecipitation using empty beads (mock; lane 3 and 4) or the TMEM129-specific 8D7 mAb (lane 5 and 6), after which immunoblotting was performed using the TMEM129-specific 4G10 mAb. Each IP lane corresponds to $20*10^6$ cells, while each input lane corresponds to $0.2*10^6$ cells. As a size reference, lysate of TMEM129-overexpressing U937 cells was included (lane 8). **(d)** CRISPR/Cas-mediated TMEM129 knockout. Lysates of U937 cells (wt, lane 1) and

TMEM129-null cells (KO, lane 2) were subjected to immunoprecipitation using the TMEM129-specific 8D7 mAb, after which immunoblotting was performed using the TMEM129-specific 13E2 mAb.

Supplementary Figure 10

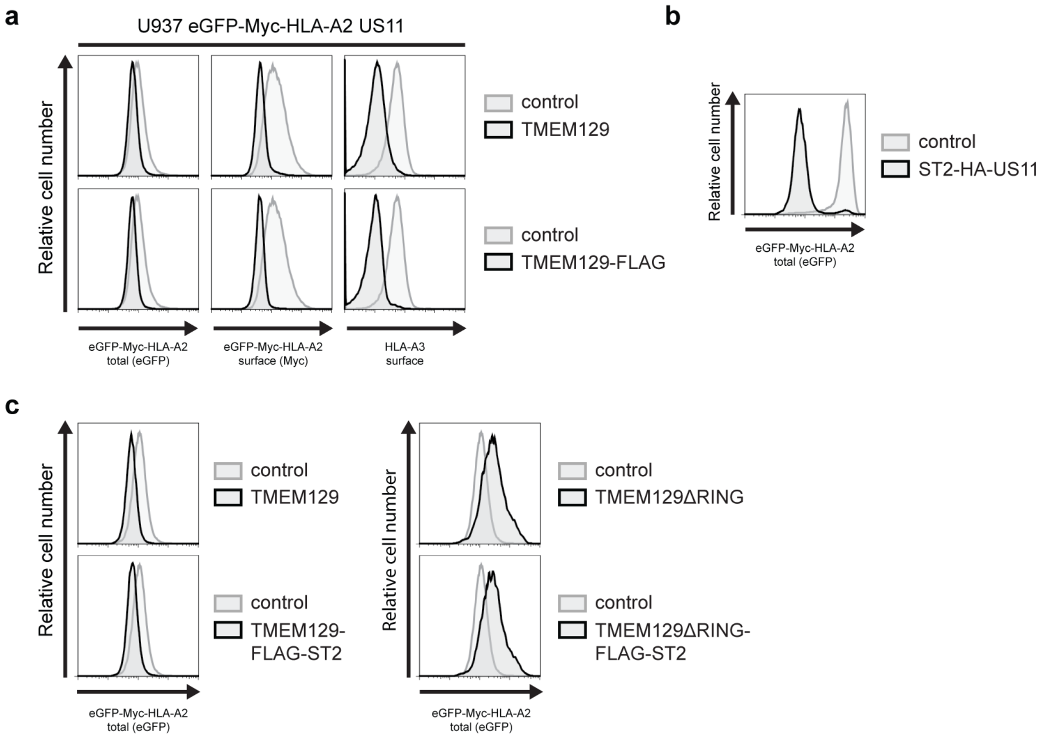

Supplementary Figure 10: Tagged TMEM129 and US11 constructs are functional.

(a) FLAG-tagged TMEM129 retains activity. Flow cytometry analysis of surface endogenous HLA-A3, and surface (Myc) and total (eGFP) eGFP-Myc-HLA-A2 in U937 eGFP-Myc-HLA-A2 US11 cells overexpressing TMEM129 (black-lined histograms, upper panels), TMEM129-FLAG (black-lined histograms, lower panels), or a control vector (gray-lined histograms, upper and lower panels). **(b)** Strep-II-HA-tagged US11 retains activity. Flow cytometry analysis of total eGFP-Myc-HLA-A2 in U937 eGFP-Myc-HLA-A2 cells expressing ST2-HA-US11 (black-lined histogram), or a control vector (gray-lined histogram). **(c)** Strep-II-HA-tagged TMEM129 retains activity. Flow cytometry analysis of total eGFP-Myc-HLA-A2 in U937 eGFP-Myc-HLA-A2 US11 cells expressing TMEM129 (black-lined histogram, left-upper panel), TMEM129-FLAG-ST2 (black-lined histogram, left-lower panel), TMEM129ΔRING (black-lined histogram, right-upper panel), TMEM129ΔRING-FLAG-ST2 (black-lined histogram, right-lower panel), or an empty vector (gray-lined histogram).

Supplementary Figure 11

Figure 2b and Supplementary Figure 1b

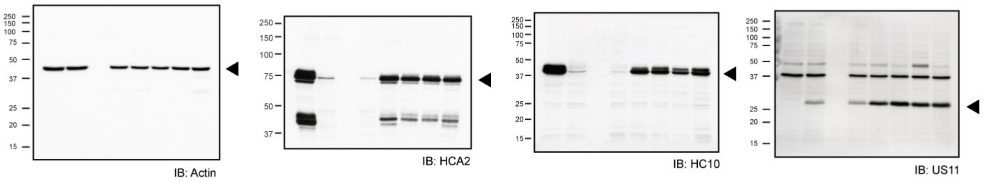

Figure 2d and Supplementary Figure 1c

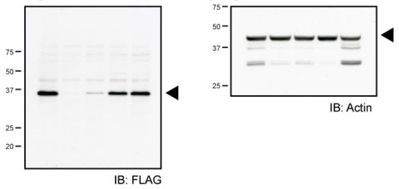

Figure 2f

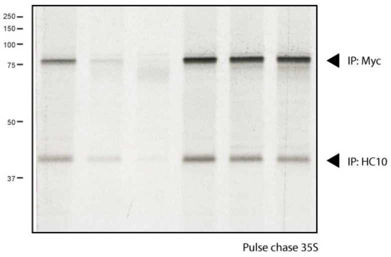

Figure 2g

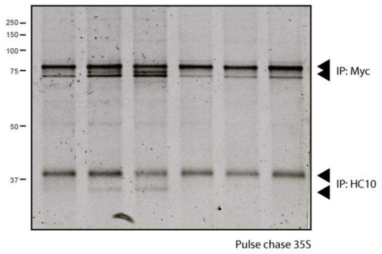

Figure 3c

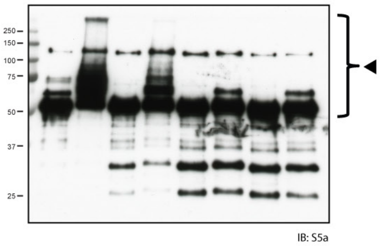

Figure 3d

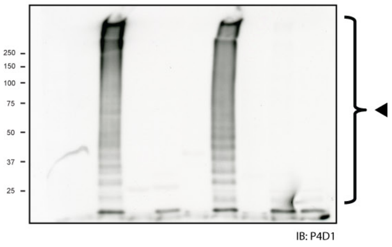

Figure 3f

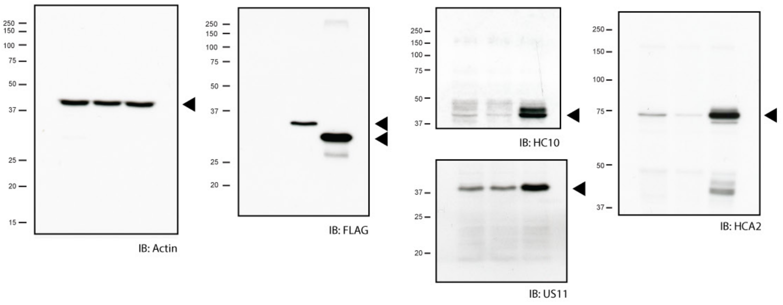

Figure 4

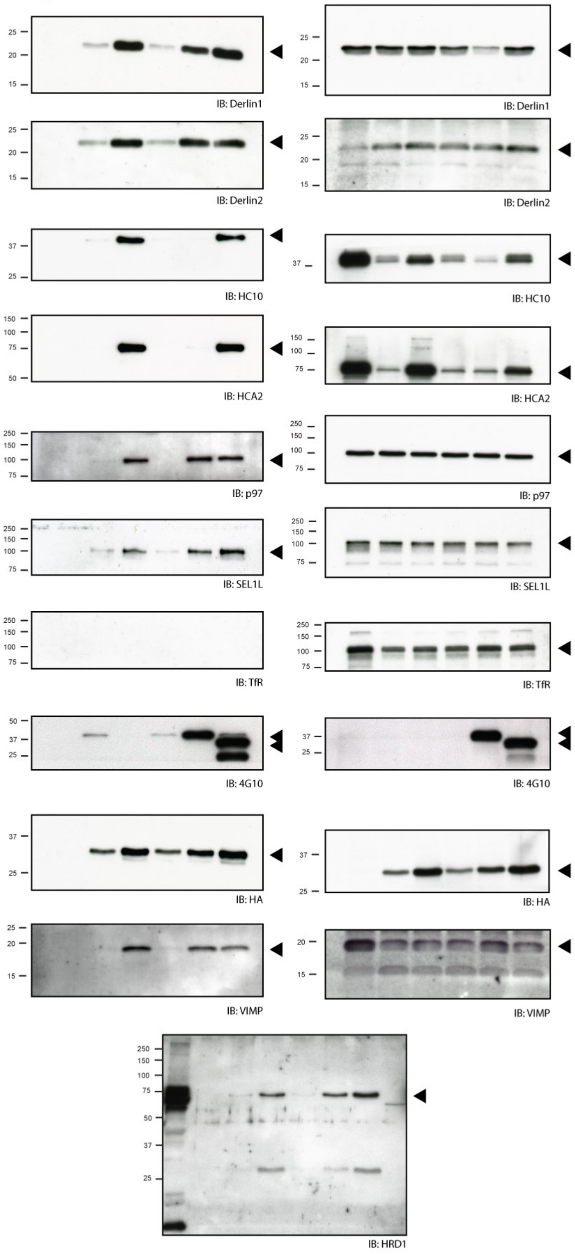

Figure 6a

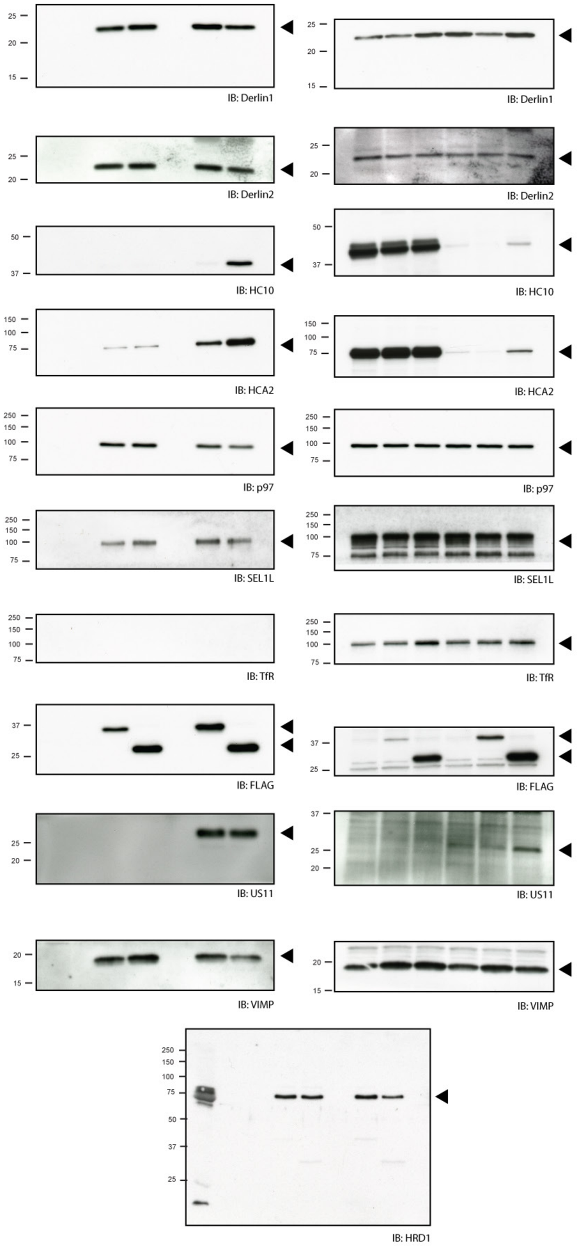

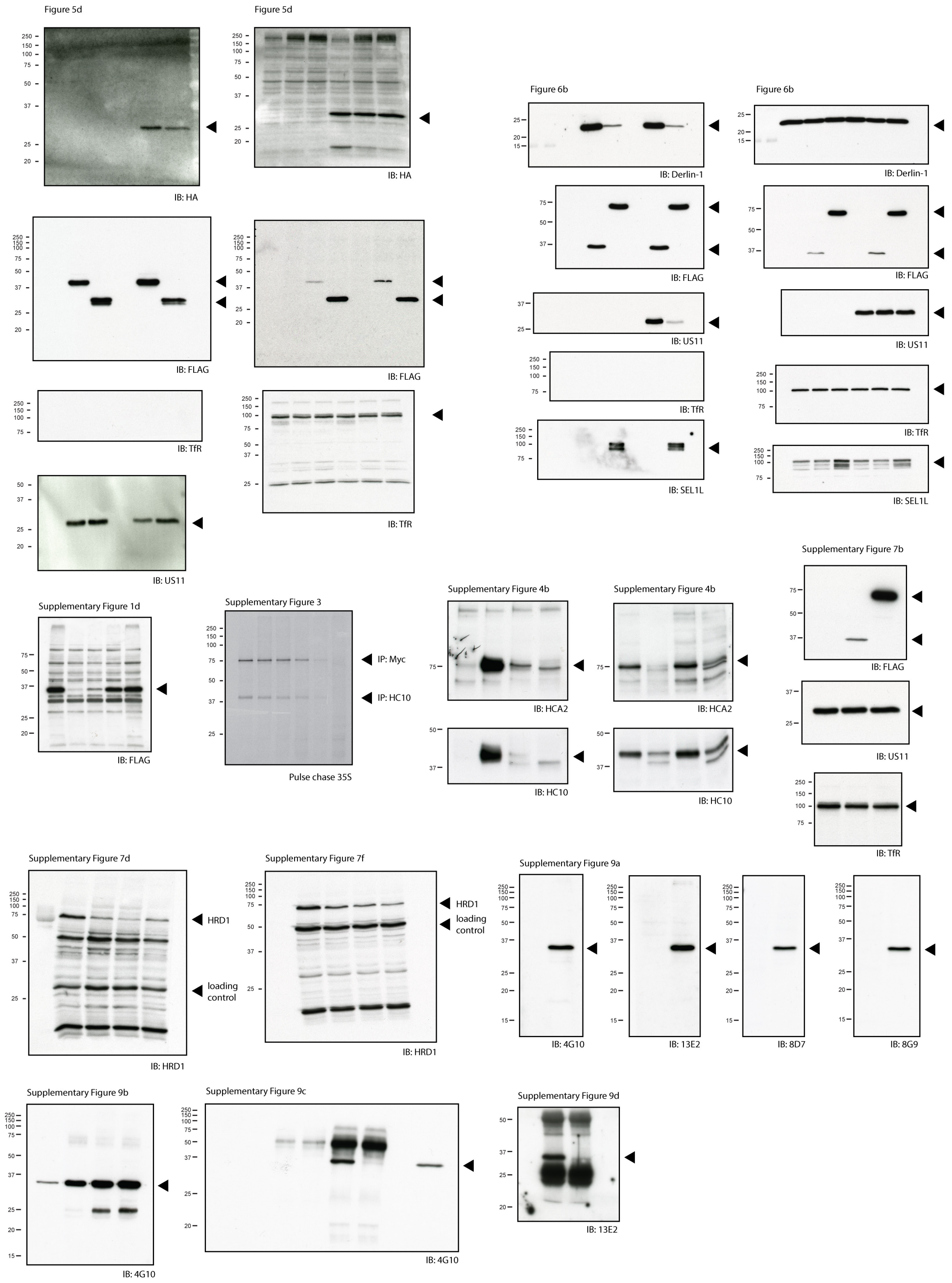

Supplementary Figure 11. Full scans Western blots. Molecular weight marker (kDa) is included on the left side of each blot. Specific bands are depicted with an arrow on the right side.

Supplementary Table 1: Sequences of shRNAs used for independent validation experiments

Name	Target	Target site in[1]	Sigma TRC number	Sigma Clone ID	Sequence shRNA	Comments
shCtrl	-	-	SHC002	Non-target shRNA Control Vector	-	
shTMEM129 #1	TMEM129	CDS	TRCN0000242616	NM_138385.3-837s21c1	CCGGTCGCCTGCATCCTGATCTACTCTCGAGAGTAGATCAGGATGCAGGCGATTTTT	
shTMEM129 #2	TMEM129	CDS	TRCN0000242617	NM_138385.3-942s21c1	CCGGTTGCCTCCTCTGTCAACACTGCTCGAGCAGTGTTGACAGAGGAGGCAATTTTT	
shTMEM129 #3	TMEM129	CDS	TRCN0000242618	NM_138385.3-1025s21c1	CCGGACGTGGGTGATGAAGGTAACCCTCGAGGGTTACCTTCATCACCCACGTTTTTT	
shTMEM129 #4	TMEM129	3UTR	TRCN0000242619	NM_138385.3-1528s21c1	CCGGCTGGCAAAGGGCTCTACTTAACTCGAGTTAAGTAGAGCCCTTTGCCAGTTTTT	
shHRD1 #1	HRD1	3UTR	TRCN0000034004	NM_032431.1-2804s1c1	CCGGTGAATGCTTAATCCCGGGAAACTCGAGTTTCCCGGGATTAAGCATTCATTTTTG	
shHRD1 #2	HRD1	CDS	TRCN0000034006	NM_032431.1-152s1c1	CCGGGCTCACGCCTACTACCTCAAACTCGAGTTTGAGGTAGTAGGCGTGAGCTTTTTG	
shHRD1 #3	HRD1	CDS	TRCN0000034007	NM_032431.1-458s1c1	CCGGGACCGTGTGGACTTTATGGAACTCGAGTTCCATAAAGTCCACACGGTCTTTTTG	
shUBE2J1 #1	UBE2J1	3UTR	TRCN0000004131	NM_016021.x-3152s1c1	CCGGGCTGAGATTGTGTGCTAGGAACTCGAGTTCCTAGCACACAATCTCAGCTTTTT	
shUBE2J1 #2	UBE2J1	CDS	TRCN0000004134	NM_016021.x-1227s1c1	CCGGCAGCTCTTATATTCCGACGAACTCGAGTTCGTCGGAATATAAGAGCTGTTTTT	
shUBE2J2 #1	UBE2J2	CDS	TRCN0000034086	NM_058167.2-408s1c1	CCGGCAAACCTCCCAGTATCTATATCTCGAGATATAGATACTGGGAGGTTTGTTTTT	
shUBE2J2 #2	UBE2J2	CDS	TRCN0000034087	NM_058167.2-358s1c1	CCGGGAAGGTGGCTATTATCATGGACTCGAGTCCATGATAATAGCCACCTTCTTTTT	
shCOPA #1	COPA	CDS	TRCN0000065269	NM_004371.2-4045s1c1	CCGGCCCTGAGTTCAAAGGTCAAATCTCGAGATTTGACCTTTGAACTCAGGGTTTTT	
shCOPA #2	COPA	CDS	TRCN0000065271	NM_004371.2-2890s1c1	CCGGGCCTTTATTGACTGTATCCAACTCGAGTTGGATACAGTCAATAAAGGCTTTTT	
shCOPB1 #1	COPB1	CDS	TRCN0000151483	NM_016451.3-2636s1c1	CCGGCCTCATGACTTCGCAAATATTCTCGAGAATATTTGCGAAGTCATGAGGTTTTT	
shCOPB1 #2	COPB1	CDS	TRCN0000151067	NM_016451.3-3110s1c1	CCGGGCCTTAAGTCTTGGAGATAAACTCGAGTTTATCTCCAAGACTTAAGGCTTTTT	Used in Suppl. Fig. 4b and 4c
shCOPG #1	COPG	3UTR	TRCN0000149699	NM_016128.3-2940s1c1	CCGGGCTTGTCCTAAATCTTGCTGTCTCGAGACAGCAAGATTTAGGACAAGCTTTTT	
shCOPG #2	COPG	CDS	TRCN0000146530	NM_016128.3-1730s1c1	CCGGGCAGGCTATATCCTAAATGGTCTCGAGACCATTTAGGATATAGCCTGCTTTTT	
shCOPZ1 #1	COPZ1	CDS	TRCN0000064998	NM_016057.1-193s1c1	CCGGCCATCGGACTGACAGTGAAATCTCGAGATTTCACTGTCAGTCCGATGGTTTTT	Used in Suppl. Figure 4b
shCOPZ1 #2	COPZ1	CDS	TRCN0000065002	NM_016057.1-249s1c1	CCGGGCAGTATAGATCTCTATTTCTCTCGAGAGAAATAGAGATCTATACTGCTTTTT	

[1] CDS, Coding sequence; 3UTR, 3'-untranslated region.

Supplementary Table 2: gRNA sequences used for CRISPR/Cas-mediated gene disruption.

Name	Gene targeted	CRISPR target site (without PAM[1])
TMEM129-gRNA#1	*TMEM129*	GACCTTCACTCTCGCCTATC
TMEM129-gRNA#2	*TMEM129*	GCACACGGCGAACACCAGAT
TMEM129-gRNA#3	*TMEM129*	GCCCAACGAGTTCCACGCGG
HRD1-gRNA#1	*HRD1*	GTGATGGGCAAGGTGTTCTT
HRD1-gRNA#2	*HRD1*	GCGGCCAGCCTGGCGCTGAC
HRD1-gRNA#3	*HRD1*	GGCCAGGGCAATGTTCCGCA
UBE2J1-gRNA#1	*UBE2J1*	GGGTCTCCATGGTGGGTCGC
UBE2J1-gRNA#2	*UBE2J1*	GTTGTAGCGGGTCTCCATGG
UBE2J1-gRNA#3	*UBE2J1*	GAATGGCACTTCACGGTTAG
UBE2J2-gRNA#1	*UBE2J2*	GTTGCACTTAAACCTCCCGT
UBE2J2-gRNA#2	*UBE2J2*	GCTGAATCCCGTTCTGGACG
UBE2J2-gRNA#3	*UBE2J2*	TATAGAGACGTCGGACTTCA
UBE2K-gRNA#1	*UBE2K*	GATTCGCTGCACCGCGATGT
UBE2K-gRNA#2	*UBE2K*	GCAATGACAATAATACCGTG
UBE2K-gRNA#3	*UBE2K*	GGATGCTGTAGTAGCAAATC

[1]PAM, protospacer adjacent motif.

Supplementary Table 3: Primer sequences used for UPR assessment.

Primer Name	Primer sequence	RefSeq	Expected band size (bp)
XBP-1 fw	CTGGAACAGCAAGTGGTAGA	NM_005080	Unspliced: 425<br>Spliced: 399
XBP-1 rev	ACTGGGTCCTTCTGGGTAGA		
GAPDH fw	CATCACCATCTTCCAGGAGC	NM_002046	404
GAPDH rev	GGCTCTCCAGAACATCATCC		

REFERENCES

1. Lebbink, R. J. *et al.* Polymerase II promoter strength determines efficacy of microRNA adapted shRNAs. *PLoS One* **6,** e26213 (2011).

2. Mali, P. *et al.* RNA-guided human genome engineering via Cas9. *Science* **339,** 823–6 (2013).

3. Rambaldi, D. & Ciccarelli, F. D. FancyGene: dynamic visualization of gene structures and protein domain architectures on genomic loci. *Bioinformatics* **25,** 2281–2 (2009).
